# ASC proneural factors are necessary for chromatin remodeling during neuroectodermal to neuroblast fate transition to ensure the timely initiation of the neural stem cell program

**DOI:** 10.1186/s12915-022-01300-8

**Published:** 2022-05-13

**Authors:** Vasiliki Theodorou, Aikaterini Stefanaki, Minas Drakos, Dafne Triantafyllou, Christos Delidakis

**Affiliations:** 1 Institute of Molecular Biology & Biotechnology, Foundation for Research & Technology Hellas, 70013 Heraklion, Crete Greece; 2 Department of Biology, University of Crete, 70013 Heraklion, Crete Greece

**Keywords:** Proneural factors, Achaete-scute complex (ASC), ASCL, Enhancers, Neural stem cell, Neurogenesis, Neuroblast, ChIP-seq, Histone marks, Notch signaling

## Abstract

**Background:**

In both Drosophila and mammals, the achaete-scute (ASC/ASCL) proneural bHLH transcription factors are expressed in the developing central and peripheral nervous systems, where they function during specification and maintenance of the neural stem cells in opposition to Notch signaling. In addition to their role in nervous system development, ASC transcription factors are oncogenic and exhibit chromatin reprogramming activity; however, the impact of ASC on chromatin dynamics during neural stem cell generation remains elusive. Here, we investigate the chromatin changes accompanying neural commitment using an integrative genetics and genomics methodology.

**Results:**

We found that ASC factors bind equally strongly to two distinct classes of cis-regulatory elements: open regions remodeled earlier during maternal to zygotic transition by Zelda and less accessible, Zelda-independent regions. Both classes of cis-elements exhibit enhanced chromatin accessibility during neural specification and correlate with transcriptional regulation of genes involved in a variety of biological processes necessary for neuroblast function/homeostasis. We identified an ASC-Notch regulated TF network that includes likely prime regulators of neuroblast function. Using a cohort of ASC target genes, we report that ASC null neuroblasts are defectively specified, remaining initially stalled, unable to divide, and lacking expression of many proneural targets. When mutant neuroblasts eventually start proliferating, they produce compromised progeny. Reporter lines driven by proneural-bound enhancers display ASC dependency, suggesting that the partial neuroblast identity seen in the absence of ASC genes is likely driven by other, proneural-independent, cis-elements. Neuroblast impairment and the late differentiation defects of ASC mutants are corrected by ectodermal induction of individual ASC genes but not by individual members of the TF network downstream of ASC. However, in wild-type embryos, the induction of individual members of this network induces CNS hyperplasia, suggesting that they synergize with the activating function of ASC to consolidate the chromatin dynamics that promote neural specification.

**Conclusions:**

We demonstrate that ASC proneural transcription factors are indispensable for the timely initiation of the neural stem cell program at the chromatin level by regulating a large number of enhancers in the vicinity of neural genes. This early chromatin remodeling is crucial for both neuroblast homeostasis as well as future progeny fidelity.

**Supplementary Information:**

The online version contains supplementary material available at 10.1186/s12915-022-01300-8.

## Background

The Drosophila genome exhibits complex and dynamic developmental chromatin and transcriptional patterns [1–6]. Due to its compact size enhancer elements are tightly spaced and utilized by many, ubiquitous and tissue-specific transcription factors (TF) [5, 7–11]. For any given cell type, specific activators turn on the relevant transcriptional program, while in parallel, repressors suppress transcription of genes related to other lineages or temporally inappropriate states, ensuring proper differentiation and maturation [12, 13].

The achaete-scute complex locus (ASC) encodes four paralogous proneural bHLH transcription factors, Achaete (Ac), Scute (Sc), Lethal of scute [L(1)sc], and Asense (Ase), which regulate central (CNS) and peripheral (PNS) nervous system development [14, 15]. They exhibit high evolutionary conservation to mammalian *ASCLs* in both sequence and proneural function [16–21]. Although prominent in neurogenesis, they also regulate progenitor cell specification and function in tissues of endodermal and mesodermal origin [22, 23]. In humans, various studies highlight their oncogenic involvement in malignancies from different germ layers [24]. Examples include small cell lung carcinomas [25], prostate tumors [26], medullary thyroid cancers [27], gastroenteropancreatic tumors [28], gliomas, grade II and grade III astrocytomas, and a subset of glioblastoma multiforme [29–33]. Also, their strong reprogramming and pioneer factor abilities [33–37] attest to their transcriptional activating potency.

Within the insects, two ancestral ASC-like proneural factors have been characterized, ASH (Achaete and Scute homolog) and Asense (Ase) [38, 39]. In many insect clades, *ASH* genes have duplicated, whereas *ase* has remained as single-copy. Drosophilids’ three *ASH* genes, *ac*, *sc*, and *l(1)sc*, exhibit a considerable degree of functional redundancy [40, 41]. In the early embryonic neuroectoderm (NE), the naïve CNS primordium, global patterning cues initiate the expression of the three ASH genes in patches of cells [42, 43]. Within these proneural clusters, cells are at a cell fate crossroad, become a neural stem cell, “neuroblast” (NB), and delaminate from the neuroepithelium or remain neuroectodermal and eventually take on the epidermal fate [44, 45]. This cell fate decision is controlled by a finely tuned interplay between ASH proneurals and Notch signaling, mostly through its E(spl)s effectors [14, 46]. Newly born neuroblasts start expressing the fourth paralogue, Ase, and other stem cell markers and divide asymmetrically to produce ganglion mother cells (GMC), which divide once to produce differentiated neurons and glia. Unlike PNS primordia, where the activity of proneural genes is required for precursor specification [15], in ASC-deficient embryos, most CNS neuroblasts delaminate, albeit at approximately 25% smaller numbers [47]. These ASC mutant NBs have restricted progeny and often die after stage 11 through a wave of apoptosis. It remains largely unknown how ASC proneurals contribute to CNS neuroblast birth and function at the chromatin level. It is noteworthy that whereas the absence of the three *ASH* genes leads to embryonic lethality, a deletion of *ase* is viable and fertile, suggesting that its NB expression is dispensable [48, 49].

Here, we have followed up on early seminal genetic work and addressed this biological process from a genomics point of view and present novel insights regarding the chromatin changes that accompany CNS neural stem cell birth in terms of global proneural binding, active histone mark deposition, and transcriptional profiles. Combining these datasets revealed a putative TF-network of proneural target genes, which are likely to comprise the forefront arsenal ensuring neuroblast functionality. Notably, ASC mutant neuroblasts undergo NE to NB transition poorly, remaining in a ‘stalled state’ characterized by a lack of timely expression of many proneural targets and, importantly, without dividing. Eventually, they overcome this arrest but cannot sufficiently sustain stem cell competence, evident by the depleted glia and neuronal population resulting in a highly hypoplastic nerve cord. Therefore, ASH proneurals appear to be largely dispensable for the NB delamination process, but are required for timely initiation of the neural stem cell program.

## Results

### Genome-wide mapping of ASH proneural binding during NB specification

**Fig. 1:**
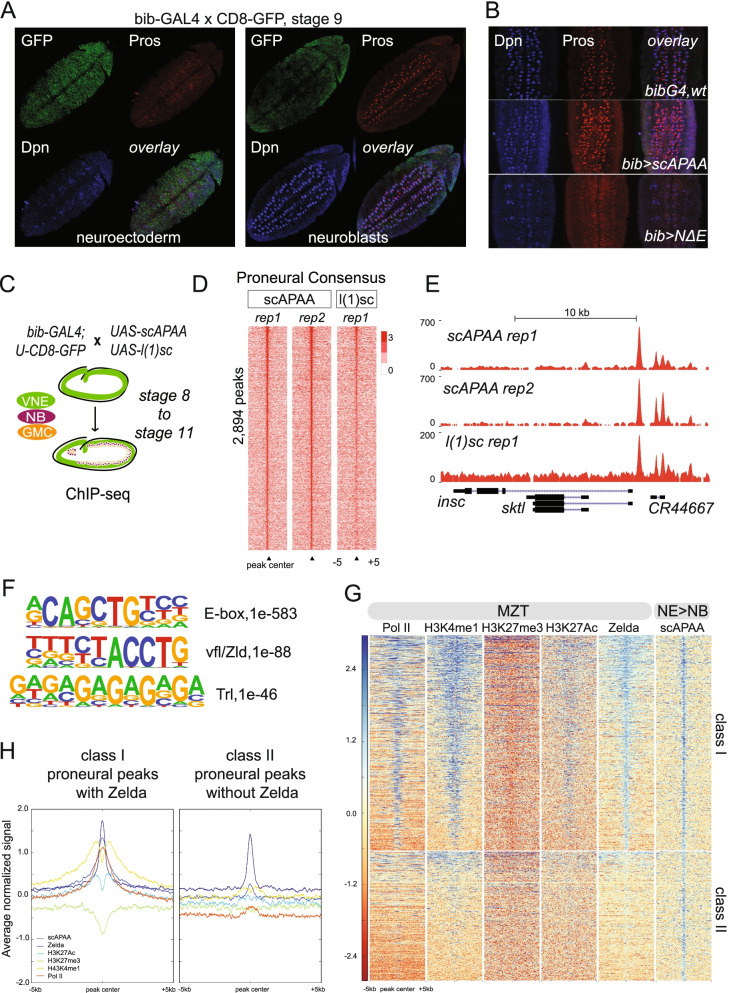
Genome-wide mapping of proneural binding in Drosophila neuroectoderm during neuroblast specification. **A** Stage 9 bib-GAL4 embryo shows GAL4 activity in the cephalic and ventral neuroectoderm. **B** Close-ups in the neuroblast field in stage 9 embryos of the genotypes shown. **C** Strategy of staged embryos used as input material to generate the ChIP-seq datasets. **D** Heatmaps of ChIP-seq normalized signal over input centered on the proneural consensus peaks. Proneural consensus peaks, Zld occupancy, and gene annotation provided in Additional file 2: Table S1. **E** Genomic snapshot at the insc gene. **F** De novo motif analysis of the proneural consensus. **G** Heatmaps of proneural, Zelda binding, histone marks and poised PolII ChIP-seq signal centered on proneural binding events, grouped in two categories: Class I occupied by Zelda earlier during MZT and Class II, Zelda-independent. **H** Average of normalized ChIP-seq signal from heatmaps in G. Proneural consensus peaks, Zld occupancy, and gene annotation are provided in Additional file 2: Table S1. Motif enrichment analysis (homer) and genomic distribution of peaks (Pavis) of Class I and Class II proneural bound regions is provided in Additional file 2: Table S2

To address the role of the ASH proneural factors, we screened a number of Gal4 lines for embryonic neuroectodermal expression and selected bib-Gal4 to express myc-tagged variants of Sc and L(1)sc for genome-wide binding and transcriptome studies. bib-Gal4 is active in the procephalic and ventral neuroectoderm from stage 8 onwards and by stage 16 GFP is detected in the ventral nerve cord (VNC) and the mature epidermis (Fig. 1A, Additional file 1: Fig. S1). During NB delamination, we detected a weak signal in the NBs (Additional file 1: Fig. S1B), indicating GFP perdurance rather than active GAL4 expression. bib-Gal4 overexpression of a wt Sc did not influence NB specification (not shown). However, induction of scAPAA, a stabilized variant [50], led to a variable, moderate increase in Dpn positive neuroblasts and Pros-positive GMCs progeny (Fig. 1B, middle panel). This subtle increase in the NB/GMC population led to mild late-stage CNS hyperplasia (Additional file 1: Fig. S1C) with varying penetrance and reduced embryonic hatching rate. On the other hand, overexpression of an extracellular domain deletion of Notch (UAS-NΔecd, abbreviated U- ΝΔE), mimicking Notch activation [51] exhibited reduced number of delaminated neuroblasts (Fig. 1B, bottom panel), severe CNS hypoplasia (Additional file 1: Fig. S1C-D) and complete embryonic lethality. These phenotypes agree with the conventional model of mutual proneural - Notch antagonism in NB specification, rendering bib-Gal4 an appropriate driver to monitor chromatin shifts during NB transition (Fig. 1C).

We focused on stage 8-mid. 11 encompassing almost the entire duration of neuroblast segregation and performed three ChIP-sequencing experiments, two against scAPAA and one against L(1)sc (Fig. 1C). A Venn diagram of called binding events among the three replicates, as well as the signal intensity heatmaps (Additional file 1: Fig. S1E), show that ScAPAA and L(1)sc bind many genomic loci commonly. We derived a consensus of common peaks between the two ScAPAA replicates (see Methods), resulting in 2,894 regions (Additional file 2: Table S1). 55% of the ScAPAA consensus was also bound by L(1)sc at the level of called peaks, possibly due to the overall weaker signal in the l(1)sc library (Fig. 1D). An example of common proneural binding is shown for the *insc* locus (Fig. 1E). We will refer to this strict, confident consensus of the two ScAPAA replicates as the ‘proneural binding consensus’ for the rest of the paper. This proneural consensus showed 27% overlap with Ac modEncode binding [52] and 12% with the Ase-DamID data [53] (Additional file 1: Fig. S1F). The limited overlap of ASH proneurals with Ase possibly reflects their expression pattern, since Ase is expressed solely in the delaminated NBs. De novo motif analysis revealed a common E-box motif in each proneural TF dataset (Additional file 1: Fig. S1F), highlighting their structural similarity. In addition, we investigated the binding co-occupancy with Daughterless (Da), a well-described proneural partner [54], and E(spl)m8, a neuroectodermal specific Notch induced E(spl) repressor that counteracts proneural/Da function, from modENCODE (Additional file 1: Fig. S1G). These global comparisons showed a 15% overlap of proneural consensus with Da and 31 % with E(spl)m8, while Da exhibited a much higher, 84% overlap with E(spl)m8 binding events. With the reservation of a technical cause for the difference, this raises the possibility that proneurals may bind mostly independently of Da and that E(spl)m8 recruitment is channeled through Da rather than proneural factors. It also agrees with the top in vivo enriched motif in all ASC proneural genomic studies, ours and others, containing the symmetric CAGCTG core (Additional file 1: Fig. S1F), rather than the asymmetric CAGGTG earlier shown to be the preferred in vitro binding motif for the Da/Sc heterodimer [55].

### Proneurals bind developmental DHS regions

Next, we evaluated the genomic distribution of the proneural binding consensus events and found high enrichments in 5kb upstream of the TSS regions, and in 5′UTRs (Additional file 1: Fig. S1H). De novo motif analysis revealed E-boxes as the primary motif identified in 73% of the proneural peak consensus, followed by the Vfl/Zelda and Trl motifs (Fig. 1F). Zelda is the pioneer factor that establishes global chromatin organization during the maternal-to-zygotic transition (MZT) [56–62], which peaks at nuclear cycle 14 (NC14), or stage 5, shortly before ASH expression in the neuroectoderm. Zelda binding together with profiles of various histone modification marks and extensive stalled PolII binding [63–65] has revealed a dynamic chromatin reorganization in preparation for zygotic transcription. We thus overlapped our proneural consensus with stage 5 Zld binding events [59] and found a 62% overlap (Additional file 1: Fig. S2A), suggesting that at these regions Zelda precedes proneural binding temporally. We used the two classes of proneural bound regions (class I with Zelda, class II without Zelda) to investigate the chromatin landscape patterns prior to proneural binding. Based on the patterns of H3K4me1 and H3K27Ac, positively associated with chromatin accessibility, the lack of the repressive H3K27me3, and the PolII signal it appears that prior to proneural binding class I target regions were nucleosome remodeled and more accessible whereas class II sites were less accessible. Subsequently, during NB specification proneurals appear to bind these loci equally strongly (Fig. 1G, H). Other than the common preferred E-box binding site, these two classes of cis-elements exhibited differences in motif enrichment analysis suggesting possible differential TF recruitment (Additional file 2: Table S2). Also, class II elements were less frequently located within a 5kb window upstream from the TSS (Additional file 1: Fig. S2B). Assignment of Class I and Class II peaks to genes identified as 1321 and 866 potential target genes, respectively. Two hundred four genes had both classes in their neighborhoods suggesting a combinatorial proneural regulation by distinct genomic regions.

Since regulatory elements correlate with DNAse Hypersensitivity Sites (DHS) [8, 11] we investigated proneural binding occurrence within stage-specific DHS and found striking overlaps (Additional file 1: Fig. S2C-D). Notably, 89% of proneural binding events were within DHS from all stages, with higher overlaps in stages 9–11 in agreement with proneural activity during the time window of NB specification. The vast majority, 98%, of class I proneural events was within DHS (Additional file 1: Fig. S2C), while class II exhibited a smaller overlap at 74% (Additional file 1: Fig. S2C). Importantly, Class I elements were open from st5 onwards, whereas Zelda-independent Class II elements were more dynamic, becoming more accessible as embryos progress from st5 to st11, perhaps as a result of proneural pioneer activity in preparation for the neural-specific transcriptional program.

### Proneurals target a plethora of genes necessary for proper NB homeostasis

**Fig. 2:**
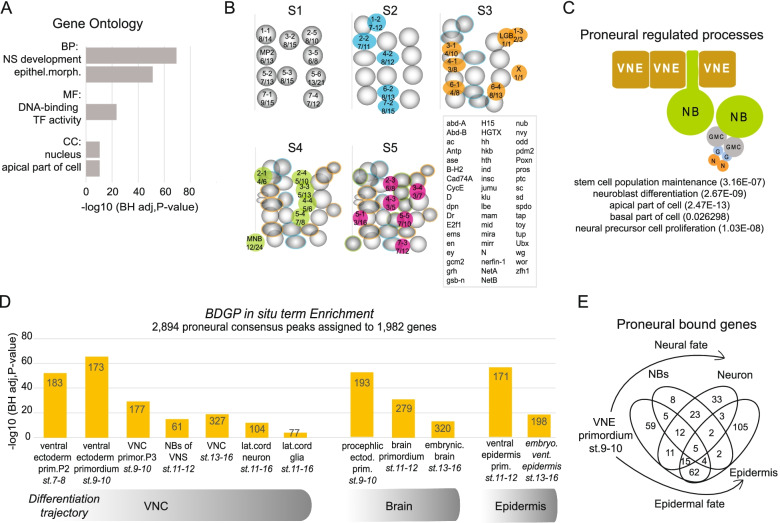
Proneurals target many genes and pathways that convey neuroblast homeostasis. **A** Gene Ontology analysis of proneural targeted genes, biological processes (BP), molecular function (MF), and cellular compartment (CC). **B** Overlap of Flybase neuroblast genes with proneural targets shown in the 5 consecutive waves of NB specification S1-S5. Numbers under the neuroblast IDs represent the number of proneural targets over the total Flybase NB-specific genes. Boxed inset lists the sum of the proneural bound neuroblast markers. **C** Proneurals regulate a holistic neuroblast program. A schematic summary of selected terms. **D** BDGP in situ enrichments of proneural target genes. **E** A venn diagram of proneural bound genes from the enriched BDGP terms in **D**. “VNE primordium” is the gene set from the second column of panel **D**; “NBs” comes from the 4th column of panel D; “Neuron” comes from the 6th column of panel **D** and “Epidermis” from the 12th column. Flymine outputs of candidate target genes (homer) near proneural peak consensus binding events; Gene Ontology, Protein Domain Enrichment, and BDGP in situ database Enrichment is provided in Additional file 2: Table S3

Next, we used the 1983 potential proneural regulated target genes in the Flymine tool [66] for downstream mining (Additional file 2: Table S3). Gene Ontology analysis (Fig. 2A) showed high enrichments for nervous system development and DNA-binding transcription factors. 53 members of the Homeobox-like domain superfamily, 69 Zinc finger C2H2-type, and 21 Helix-loop-helix DNA-binding domain superfamily genes were among the proneural targets, suggesting proneural regulation of a broad network of transcription factors.

Next, we extracted from Flybase [67] genes associated with each specific neuroblast and found proneural binding in 53 out of 98 neuroblast markers, of all five waves of neuroblast specification (Fig. 2B). Besides genes that presumably provide neuroblast identity (stemness), many different processes are needed for proper NB function: delamination; establishment of cytoplasmic asymmetry, expression and correct segregation of pro-differentiation factors, self-renewal and proliferation through multiple asymmetric divisions, and temporal progression of progeny types [14, 68]. Notably, proneural target genes fell in all the above-mentioned processes. For instance, the known stem cell identity markers *wor*, *dpn*, *scrt, klu,* the temporal genes *hb*, *Kr*, *nub*, and *grh* [69]; genes encoding myosin contractile machinery important for delamination, like *zip*, *sqh*, *Rok*, and *Rho1* [70]; the cell cycle genes *cycE*, *E2F1*, and *stg*; and members of apico-basal polarity organizing Par complex (*baz*), Pins complex (*insc,loco,mud,cno*), the Centrosome organizing center (*ctp, mud*), and the basal compartment (*mira, brat, pros*) [71]. Thus, proneurals appear to regulate many biological processes needed for neuroblast homeostasis (Fig. 2C).

In addition, we investigated the expression patterns of the proneural-targeted genes using the BDGP in situ RNA database integrated in the Flymine tool (Fig. 2D, Additional file 2: Table S3). We found that many target genes express in the ventral ectoderm primordium, but also in the brain, VNC, midline, and sensory primordia at the time of neural specification. We also found binding near genes expressed in later developmental stages, in differentiated cell types such as neurons and glia, also supported by the GO enrichments in neuron differentiation [GO:0030182] and axonogenesis [GO:0007409] (Additional file 2: Table S3). A Venn diagram of proneural-bound genes, expressed in the ventral ectoderm, NB, VNC neurons, and epidermis (BDGP), showed common as well as unique genes per cell type (Fig. 2E). Thus, we speculate that besides orchestrating the neuroblast program, during the NE to NB transition, proneurals may remodel chromatin in preparation for more committed differentiation states.

### Proneural binding enhances chromatin acetylation

**Fig. 3:**
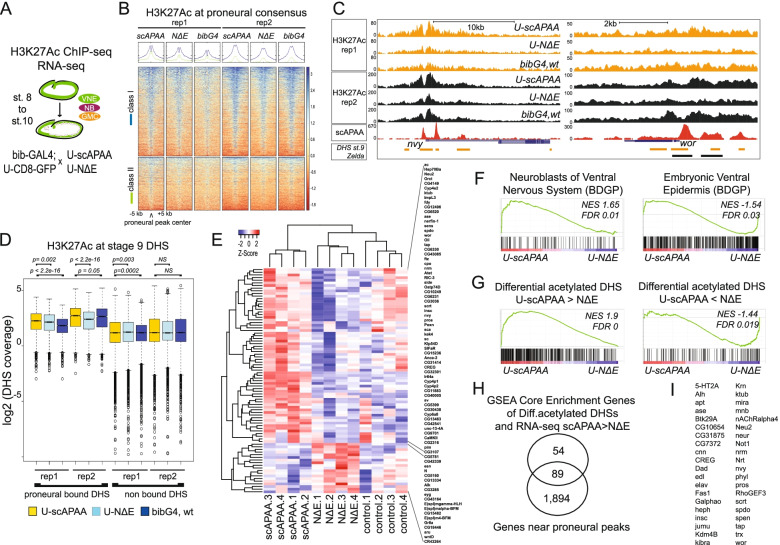
Proneural mediated chromatin changes correlate with transcriptional output during early neurogenesis. **A** A schematic representation of the strategy used to generate H3K27Ac ChIP-seq datasets and RNA-seq profiling. **B** Heatmaps of H3K27Ac ChIP-seq signal centered on Class I and Class II proneural peaks. **C** Genomic snapshots at the *nvy* and *wor* loci. **D** Boxplots of normalized H3K27Ac signal in stage 9 DHS sites from modENCODE. 2090 DHSs with proneural binding (left), not proneural-bound DHS 14,127 (right). Statistics performed with Wilcoxon rank-sum tests. **E** Differentially expressed genes from RNA-seq in scAPAA versus NΔE embryos FDR 0.2 (*n* = 4). **F** Gene Set Enrichment Analysis (GSEA) of RNA-seq data reveal enrichment for BDGP “VNC neuroblasts” with scAPAA>NΔΕ genes and “ventral epidermis” classes with the scAPAA<NΔΕ genes. **G** GSEA of differentially acetylated st.9 DHSs in scAPAA. vs. NΔΕ embryos (scAPAA> NΔΕ left, scAPAA<NΔΕ (right) with the ranked genes from the RNA-seq of the same comparison. **H** A Venn diagram of the GSEA Core Enrichment Genes from **G** (left panel) with potential target genes of the proneural consensus binding events. **H** List of selected 40 genes from the intersection in **H** with proneural binding and combined RNA-seq and differential acetylation. Differential acetylation testing on stage 9 DHS regions in wt bibG4, bib>UscAPAA, and U-NΔE embryos (*n* = 2) is provided in Additional file 2: Table S4. RNA-seq edgeR analysis output provided in Additional file 2: Table S5

Next, we asked whether proneural activity affects chromatin organization in terms of enhancer remodeling and transcriptional output. For this reason, we generated replicated RNA-seq experiments and H3K27Ac ChIP-seq datasets from staged embryos (Fig. 3A). We restricted the time window for these experiments by 1 h (stage 8-mid 10) compared with the proneural ChIP-seq datasets, to ensure monitoring of the initial process of NE➔NB specification and dilute out possible signals from more differentiated cell types. We performed various analyses to integrate expression and acetylation data with proneural chromatin binding. First, we focused on the proneural peak consensus and found a higher H3K27Ac signal in the U-scAPAA embryos, in both class I and class II regions (Fig. 3B). Of note, Class II elements, which at NC14 exhibited overall low accessibility, in st.8–10 exhibited increased H3K27Ac signal (compare the averaged signal in NC14 Figs. 1H, 2 and 3B), suggesting that they become more active as development progresses. We used the Zelda peaks not bound by proneurals as a negative control dataset and showed that the scAPAA-mediated increase in H2K27Ac deposition compared against the wt BIB and NΔE genotypes, was more statistically significant on the class I and class II proneural consensus peak datasets than on the control Zelda peaks (not bound by proneurals) (Sup.Fig. S2E). Genomic snapshots at *nvy* and *wor*, two bona fide neuroblast markers [72, 73] are representative examples of regions that exhibited both H3K27Ac proneural increase and NDE repression (nvy) or mostly NDE repression (wor) (Fig. 3C). In accordance, analysis of H3K27Ac mark on st9 DHS sites, revealed an increased signal in the proneural-bound DHS regions (left panel) compared to the non-bound DHSs (right panel) (Fig. 3D). This indicates that Drosophila ASH proneurals enhance active chromatin conformation, consistent with the pioneer function of mammalian homologs [36, 37].

We subsequently asked which DHSs were most affected in H3K27Ac deposition in scAPAA vs. NΔE conditions, as a way to monitor the neuroblast versus epidermal cell fate selection during lateral inhibition. We performed differential analysis enrichment per genotype using the RPKM counts of each H3K27Ac library (see the “Methods” section) on all DHS st.9 regions (Sup. Table S4). At FDR 0.2, we found 311 DHSs with differential H3K27Ac signal in scAPAA.vs.ΝΔΕ, 80 of which were also differential in scAPAA.vs.BIB and 31 also in NDE.vs.BIB. None was common in all three comparisons. These 311 genomic sites were near 284 genes, enriched in ventral ectoderm and nervous system-related genes (not shown), similar to the proneural consensus distributions of Fig. 2D. However, only 16%, (49 sites), of the affected DHSs in the scAPAA vs. NDE comparison coincided with proneural binding. The remaining 262 not-bound DHSs were close to 255 genes, which showed a 24% overlap at the gene assignment level with the proneural-consensus genes, indicating that proneural binding might have broader effects outside its binding element. Alternatively, these differentially acetylated DHSs may represent cis-elements regulated by Notch signaling independently of ASH activity.

### Combination of transcriptome and chromatin profiling reveals putative core regulators of neural stem cell function

To identify the transcriptional changes that accompany neural selection, we performed RNA-seq expression profiling. In the differentially expressed genes (DEG) between the U-scAPAA and U-NΔE embryos (FDR<0.2, *p*<0.0025) (Fig. 3E and Additional file 2: Table S5) there were many neurogenesis related transcription factors. Indeed, Gene Set Enrichment Analysis (GSEA) of the ranked genes from this comparison against BDGP annotations clearly mirrored the neural versus epidermal fate specification that proneurals and Notch favor respectively (Fig. 3F). In addition, we found significant enrichments of scAPAA>NΔΕ upregulated genes with the differentially acetylated DHSs in scAPAA>NΔE (1257 DHSs at *p*<0.05) (Fig. 3G), as well as with the class II proneural binding events (Additional file 1: Fig. S2F). Additional GSEA tests with the differentially acetylated DHS from scAPAA and NΔE versus BIB control comparisons, also correlated significantly with the ranked gene expression from the RNA-seq comparisons (Additional file 1: Fig. S2G-I). These correlations demonstrate that the regulatory elements filtered out from the above integrative genomics analyses are transcriptionally relevant, suggesting that the proneural-mediated activation is counteracted by the NΔE repression at the chromatin level during embryonic neurogenesis on these loci. To expand on this observation, we took the 143 Core Enrichment Genes from the GSEA presented in Fig. 3G (left panel), which exhibit both positive transcriptional regulation and enhanced acetylation in their neighboring DHSs in ScAPAA vs NΔE, presumably genes favoring the neural differentiation path, and overlapped them with the putative target genes from the proneural binding consensus peaks (Fig. 3H). We found that 89 genes (62%) also bore proneural binding events. Fig. 3I shows a selected panel of 40 genes, including many TFs known to regulate neurogenesis but not associated with proneural regulation to date. For example, in this high-confidence gene set we find *Alh*, *ase*, *apt*, *edl*, *jumu*, *Neu2*, *tap*, *pros*, *scrt*, *wor*, and *nvy*, known to act in the CNS, PNS, and midline. Thus, this TF network regulated by proneural and Notch interplay could be the initial battery of factors required to sustain neural precursor functionality.

### ASC mutant neuroblasts are temporarily stalled and devoid of stem cell identity markers

*ASC* null (*Df(1)scB57* or *Df(1)260.1*) embryos are known to display a drastic reduction of mature neurons, whereas at earlier times they only show a mild reduction in delaminated neuroblasts [47]. However, it has not been documented how these mutant NBs behave. Taking advantage of our genomic data, we launched a detailed analysis of NB-related transcription factors in wt vs ASC deficient embryos. We selected TFs whose genes are near proneural binding peaks; some of them also show differential RNA expression or histone acetylation in our experiments (Additional file 2: Table S6 for a complete description). This TF list consists of the NB-specific TFs Dpn, Sna, Wor, Klu, and a set of “NB and GMC” expressed factors, broken down in three subgroups: (a) the pan-NB, pan-GMC markers Esg, Scrt, Nvy, and Pros; (b) the temporal factors Hb, Kr and Grh, expressed in temporal waves in the NBs and more persistently in the GMCs and neurons born from the positive NBs, and (c) the “mostly-GMC” markers Nerfin-1, Oli and Tap, which are also transiently expressed in NBs or a subset thereof. To these we added the segment polarity markers En, Odd and Mirr, expressed in the ectoderm and underlying NBs, to help us identify specific NB rows; these, too, are located near proneural binding events. In all, we evaluated the expression of 17 TFs.

**Fig. 4:**
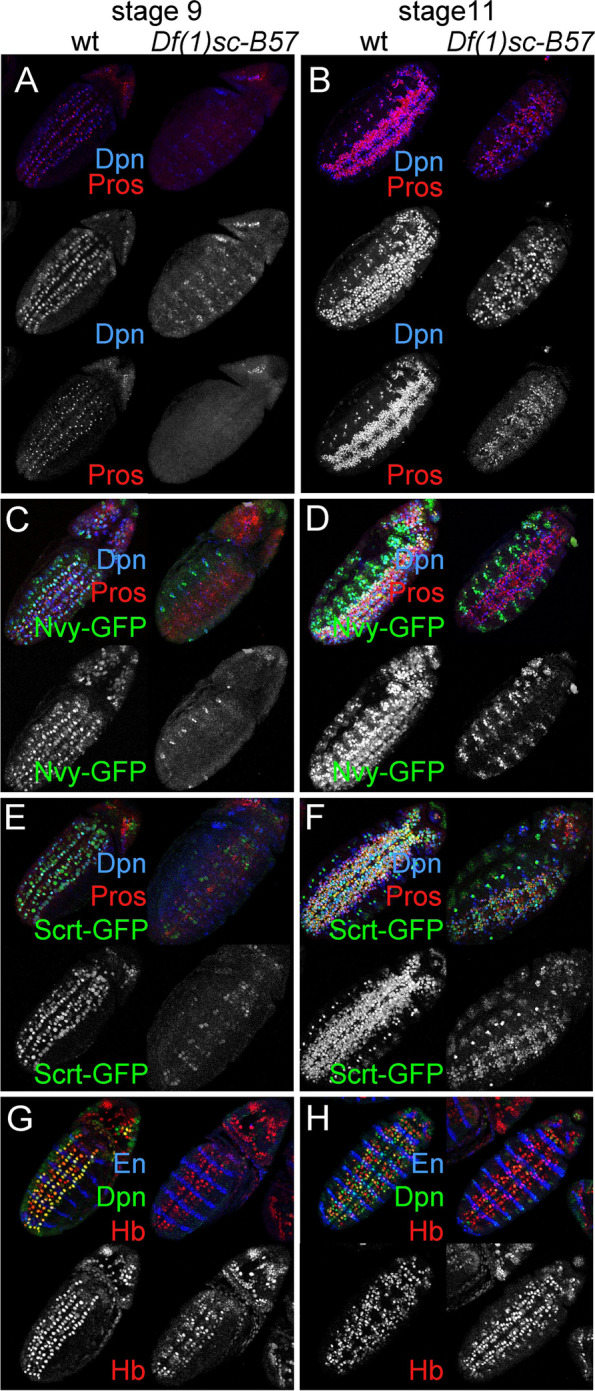
ASC mutant neuroblasts are temporarily stalled and devoid of stem cell identity markers. **A** Stage 9 wt neuroblasts (left panels) express Dpn and have divided to generate Pros positive GMCs. In *Df(1)scB57* embryos (right panels) neuroblasts do not express Dpn and have not yet divided to produce GMCs. The weak Dpn signal in the mutant embryo comes from the NE layer above the delaminated NBs. **B** In stage 11, mutant neuroblasts have rebounded in Dpn expression and cell divisions to produce GMCs. The sparse Dpn and Pros positive cells outside the broad band of the VNC are PNS precursors, which are also strongly reduced in the ASC mutant. **C** Nvy-GFP is absent in mutant neuroblasts during stage 9. Remaining expression comes from more laterally positioned PNS precursors, **D** Nvy expression does not rebound in mutant neuroblasts at st 11. **E** Scrt is lost or very weak in mutant neuroblasts at st 9. **F** Scrt expression rebounds in stage 11 *Df(1)scB57* neuroblasts and GMCs. **G** Stage 9 *B57* mutant neuroblasts, not expressing Dpn, express hunckback (Hb). Notice the lack of Hb positivity on the engrailed (En) stripe and the lateral NB column in the mutant. **H** In stage 10 *B57* mutants, Hb is seen over the En stripe. A summary of genomic characteristics of selected proneural TF targets tested is provided in Additional file 2: Table S6

A first striking observation was that during the early stages of neurogenesis (embryo stages 8-10 or NB delamination waves S1-3) mutant NBs are stalled. We found that Dpn (Fig. 4A), Wor (Additional file 1: Fig. S3A-B), Nvy (Fig. 4C), Scrt (Fig. 4E), Nerfin1 (Additional file 1: Fig. S4B), Klu (not shown), and Oli (Additional file 1: Fig. 4SA, middle panels) were undetectable or severely underexpressed in mutant NBs. In contrast, Mirr, Odd, En, Esg, Sna, Hb, and Kr were expressed but displayed mild defects (Additional file 1: Fig. S5, discussed below). As an example, in stage 9 mutant embryos there was no robust Hb staining on the En stripe or the lateral NB column (Fig. 4G and Additional file 1: Fig. S5B). This irregular NB marker profile suggests defective neural stem cell fate specification.

Concomitant with this cell-fate defect, we observed that mutant NBs were mitotically stalled. Wt NBs embark on GMC-producing divisions soon after delamination and by early st10 a large number of Pros-positive GMCs are present. Significantly, ASC mutant embryos contained no GMCs during this time window (Fig. 4A), suggesting a NB cell cycle arrest. This was also demonstrated by the lack of pH3 mitotic events in mutant NBs, compared to their wt counterparts. (Additional file 1: Fig. S3B). To address NB divisions further, we used the UAS-FUCCI, a dual expressing GFP-E2F1 and RFP-CycB system, that allows cell cycle monitoring by fusing cell-cycle specific degrons to fluorescent proteins [74]; GFP-E2F1 is degraded at the S-phase, whereas RFP-CycB is degraded at the end of M and G1. Consistent with bib-Gal4 activity specifically in the NE and the rapid onset of NB mitoses, wt NBs showed little or no accumulation of FUCCI signal (Additional file 1: Fig. S3C). *ASC*^-^ NBs, however, accumulated both these markers demonstrating a G2/M arrest, suggesting that after delamination they retained the NE-expressed FUCCI signal since they had not divided yet (Additional file 1: Fig. S3D). These results show that *ASC* deficient neuroblasts undergo NE to NB transition poorly, as they do not proliferate, nor do they initiate expression of a large part of the neural TF program on time, suggestive of incomplete programming (Additional file 1: Fig. S3E).

### Mutant NBs exhibit a partial rebound of the NB/GMC program by stage 11

Despite this early developmental arrest, starting at late stage 10/early 11, we observed a gradual rebound in NB marker expression, accompanied by the initiation of NB mitoses. By late stage 11, ASC mutant NBs had started expressing Dpn (Fig. 4B), Scrt (Fig. 4F), Oli (Additional file 1: Fig. S4), Hb (on the En stripe) (Fig. 4H and Additional file 1: Fig. S5B), Wor and Klu (not shown). The only marker that never rebounded was Nvy (Fig. 4D). In parallel, many Pros-positive GMCs were born (Fig. 4B), that expressed, Scrt (Fig. 4F), Oli (subset, Additional file 1: Fig. S4A), and Nerfin-1 (subset, Additional file 1: Fig. S4B), Esg, Hb (subset) and Kr (subset), but not Nvy (Fig. 4D) or Tap. Tap is normally expressed in a large subset of GMCs from late st10 to st15/16 (Additional file 1: Fig. S4C). In *ASC*^-^ GMCs, Tap showed a prolonged delay and eventually turned on by stage 13 (not shown). In other words, the *ASC*^-^ GMC profile also seems impaired.

### ASC proneural TFs are largely dispensable for the delamination process and the temporal cascade

Even though ASC mutant NBs displayed a pronounced delay in the onset of their developmental program and GMC generation, their delamination from the ectoderm did not appear to be greatly affected (Additional file 1: Fig. S5). We initially saw this by the presence of large delaminated cells at stage 9/10 using the pan-neuroectodermal plasma membrane marker Nrt (Additional file 1: Fig. S5A) and verified it with additional NB markers. Most NBs had ingressed and expressed Hb robustly at the S1 delamination wave; even those in the lateral column (NBs 2–5, 3–5, 5–6) were faintly detectable (Additional file 1: Fig. S5B). The ones in the En stripe, which did not express Hb at S1, showed Hb expression at the time of S2 (Additional file 1: Fig. S5B). NBs 6-2 and 7-2 were almost always distinguished by En/Hb positivity at S2 delamination, NB7-1 became Hb-positive towards st.10 (Additional file 1: Fig. S5B, right panel), while we confidently identified 7-4 only by Sna/En co-expression (Additional file 1: Fig. S5C). NB1-1 and 2-5 were detected in mutant embryos by Odd (Additional file 1: Fig. S5D) and Mirr (Additional file 1: Fig. S5E). Mirror-GFP also marked the GP in stage 10 (Additional file 1: Fig. S5E). However, the mutant Repo-positive GP had not divided in late stage 10, possibly due to the initial stalling after delamination. We did not detect any Odd-positive MP2 NBs (Additional file 1: Fig. S5F), although we could detect a presumptive MP2 by Hb staining (Additional file 1: Fig. S5B). Overall, this marker analysis confirms the presence of many early NBs in the *ASC* mutant, although the complement of NB-specific markers is disturbed.

Next, we tested the temporal patterning of NBs in the ASC deficiency. As described above, Hb was turned on almost normally at S1/S2 and turned off in most NBs normally at early st11, soon after Dpn rebound. As in the wt, Kr expression in the mutant started early and persisted in NBs into late st11, whereas the late marker Grh was turned on normally at st13 (Additional file 1: Fig. S5G) and persisted until the end of embryogenesis (not shown). Therefore, to the best of our knowledge, the beginning and the end of the temporal cascade do not seem grossly perturbed in ASC^-^ NBs.

### *ASC* null neuroblasts are defectively programmed and produce impaired progeny

We have heretofore shown that ASC mutant NBs are born in a stalled state, followed by a rebound of many stem cell markers and a concomitant delayed start of mitotic activity. Despite this rebound in mutant NB identity, late embryos are severely hypoplastic, with fragmented nerve cords. Staining with axonal markers revealed a complete lack of the three VNC longitudinal nerve tracts and severe defects in intersegmental/segmental nerves (Additional file 1: Fig. S6A), see also [47, 75]. Axonogenesis is normally guided by communication cues between neurons and glia from the CNS, PNS, and midline [76–81]. Glia play a crucial role both in prefiguring axonal paths and in providing trophic support to neurons. This is evident in glia-depleted, *gcm* mutant embryos [82], where longitudinal nerve tracts also fail to develop similar to the ASC mutants. We found a diminished glia population in late ASC embryos. This was more evident in the abdominal segments, by an at least 70% reduction in Repo positive glia (Additional file 1: Fig. S6B). Specifically, the two characteristic continuous columns of longitudinal glia lining the dorsal side of the developing nerve cord from st13 onwards were depleted. The Repo/Mirr positive longitudinal glioblast progenitor (GP) was present in many hemisegments earlier (st.10/11) (Additional file 1: Fig. S5E); suggesting that the mutant GP fails to produce the correct glia progeny. We asked whether other NBs may also display similar defects in lineage production.

**Fig. 5:**
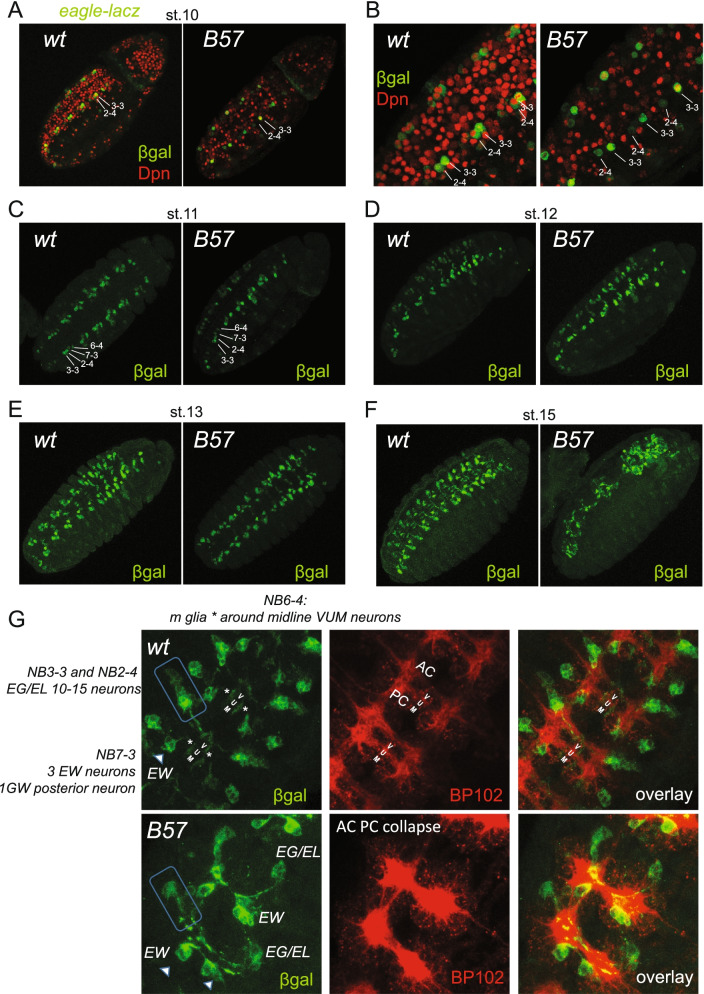
ASC proneurals contribute to late-born NB identity and progeny fidelity. **A** Eagle-lacz expression in stage 10 embryos. In wt the NB 2-4 and 3-3 is clearly visible, expressing eagle and Dpn robustly. In B57 mutant embryos 2-4 in not clearly seen, as it expresses eagle weakly. **B** Close-ups of embryos in **A**, show that the 2-4 neuroblast is also temporarily stalled since it does not express Dpn. **C** Eagle-lacZ expression shows that at stage 11 all four positive neuroblasts have delaminated in Df(1)scB57. **D** Stage 12 embryos do not show major differences in eagle-lacZ positive cells. **E** By stage 13, the medial glia, progeny of 6-4, that move towards the midline are absent or have lower expression in the Df(1)scB57. **F** By stage 15 the nerve cord shows severe disorganization in mutants. **G** Stage 15 VNC close-ups show an Anterior-Posterior Commissural axonal collapse, no medial glia (asterisks in wt), a diminished EG/EL neuronal population (boxed), an EW neuron missing (left arrowhead) whereas the other EW (right arrowhead) is sending its axon laterally instead of posteriorly

We investigated specific, well-described NB lineages. It is known that the MP2 progeny, the two pioneer sibling vMP2/dMP2 neurons, are absent or mis-specified in ASC mutants [83, 84]. We confirmed this by imaging odd-GFP in stages 14-15: *ASC*^-^ displayed mostly one or two Odd-positive cells per segment, the MP1, and its progeny, at a time when wt embryos normally contain a quartet of Odd-positive cells, the two MP1 progeny and two dMP2 neurons (Additional file 1: Fig. S6C). This indicates that mutant MP2 did not divide or, if it did, it gave aberrant progeny. We next used Eve/FasII staining, to identify the aCC/pCC sibling neurons, the two first neurons to express Fas2 at late st10 in the wt. Although their parental NB1-1 neuroblast was robustly seen in st9/10 ASC- embryos (Additional file 1: Fig. S5A, B, D), we never detected the Eve/ Fas2 positive aCC/pCC pair at st10 or 11 in *ASC*^*-*^ embryos (Additional file 1: Fig. S6D). We could however see an unusual Fas2-positive/ Eve-negative pattern at the position where aCC/pCC should lie. Only one medial Eve positive was consistently observed, presumably the RP2 motor neuron (S2 NB4-2 progeny), which survived in almost all neuromeres until late embryogenesis (Additional file 1: Fig. S6D, E). Eve immunoreactivity also reveals the progeny of the S1 NB7-1 and the S4 NB3-3 neuroblasts, producing the U- and EL- neurons respectively (Additional file 1: Fig. S6E, right panels). Most hemineuromeres contained a reduced number of ELs in mutant st. 13-14 embryos; we also noted several instances where ELs were absent, despite the presence of NB3-3 in all hemineuromeres of st.10-11 embryos (see below Fig. 5). In contrast to ELs, which were frequently observed, U neurons were almost never seen; in rare instances, one or two Us were detected per embryo (Additional file 1: Fig. S6F). Therefore, both NB3-3 and 7-1 are unable to properly execute their lineages in an *ASC*^*-*^ background.

Other than the EL neurons and their parental neuroblast, (NB3-3 S4), all above progeny comes from precursors specified at the early S1–S2 waves of neurogenesis. We wondered whether ASC mutants also exhibit defects in neurons/glia born from later NB waves during late st10-11, a time when mutant NB activity has rebounded. eagle-lacZ is a marker of four late NBs and their progeny, (S3:NB6-4, S4:NB2-4, and NB3-3 and S5:7-3) [85]. At stage 10/11, a time when Dpn starts rebounding in ASC^-^ NBs, we observed that NB2-4 and NB3-3 are present, but many (53%) are stalled in Dpn expression (Fig. 5A, B), which suggests that late delaminating ASC- NBs also exhibit an initial identity defect. At later times eg-lacZ/Dpn-positive NBs were present in most neuromeres (Fig. 5C–F) but they produced variably depleted progeny with deformed axonal projections, accompanying an anterior to posterior commissure (AC-PC) collapse (Fig. 5E–G). Collectively, these observations demonstrate that ASC deficient NBs, both from early and late phases of specification, cannot sustain correct progeny differentiation, suggesting that the severe CNS hypoplasia is the result of inherently defective neural programming rather than delamination defects.

### *ase* can substitute for the *ASH* genes to initiate the neural program in the neuroectoderm

**Fig. 6:**
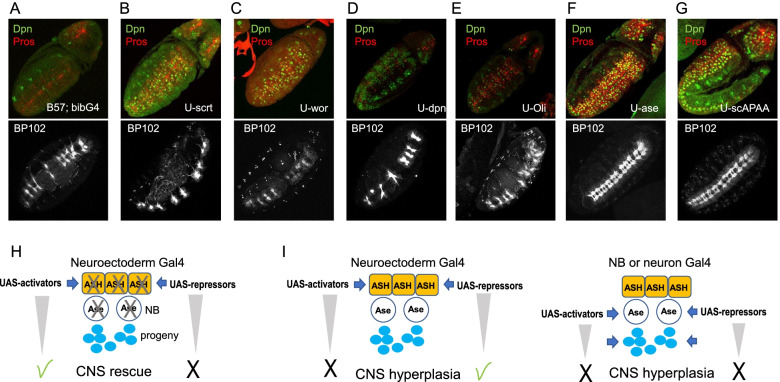
ASC loss is hard to compensate. Early and late rescue phenotypes of neuroectodermally (bibGal4) induced proneural targets in the Df(1)scB57 background. Early embryos (top row) stained with Dpn and Pros, late embryos (bottom row) stained with the axonal marker BP102. **A** Df(1)scB57; bibGal4 with no UAS transgene. **B**–**G** as in **A**, plus **B** UAS-scrt, **C** UAS-wor, **D** UAS-dpn, **E** UAS-Oli, **F** UAS-ase, **G** UAS-scAPAA. **H** Model of ability of selected genes to rescue the Df(1)scB57 neuronal hypoplasia. **I** Model of ability of selected genes to induce neuronal hyperplasia in the wt background. Activators refer to ASC genes; repressors refer to Snail and Hes family genes. Effect is shown by a check mark; lack of effect by X

We next investigated whether any of the downstream proneural targets revealed by our genomic experiments would be able to rescue the neurogenesis defects of the *ASC* deficiency, if transgenically provided using the neuroectodermal driver bib-Gal4 (Fig. 6). We tested UAS-scrt, UAS-wor, UAS-dpn, and UAS-Oli, four of the proneural targets that showed a delayed onset of expression in the absence of the ASC. None of these was able to rescue NB stalling at st9. We observed a detectably earlier rebound of NB activity at early st10, evident by the earlier Dpn expression and the emergence of Pros+ GMCs (Fig. 6B–E, top panels). Nonetheless, this slight NB rescue was not able to improve the severe late hypoplastic phenotype (Fig. 6B–E, bottom row), suggesting that these factors are not capable of activating the full neurogenic program in the absence of ASC genes. In contrast, induction of UAS-scAPAA or UAS-ase led to a vast improvement to the stalling defect of NBs (Fig. 6F, G, top), which now expressed Dpn and started dividing normally at st9. At later stages, the VNC was almost complete with only minor constrictions (Fig. 6F, G, bottom).

Therefore, re-instating proneural expression in the neuroectoderm can greatly rescue neurogenesis demonstrating that the ASH and Ase proteins have equivalent activities, despite their distinct expression patterns. To clarify this further we used the *Df(1)sc19 ASC* deficiency, which deletes *ac*, *sc*, and *l(1)sc*, but spares *ase*. In this background, NB stalling was still evident during stage 9 (Additional file 1: Fig. S7A). Ase itself also exhibited a small delay in expression; however, its expression preceded Dpn (Additional file 1: Fig. S7B) and Pros (not shown), both rebounding soon after Ase expression by early stage 10 (Additional file 1: Fig. S7C), earlier than in *Df(1)scB57*. The late CNS hypoplasia was also improved in *Df(1)sc19*. The population of glia was richer (Additional file 1: Fig. S7D) and the aCC/pCC pioneer neuron pair was sometimes present (not shown). The VNC had fewer neuromere gaps, as reported by [75], although the wt pattern of three Fas2-bearing longitudinals was not fully restored (Additional file 1: Fig. S7E). Therefore, the endogenous expression of Ase in the delaminated neuroblasts can greatly improve NB functionality (sc19 vs. B57), but not as efficiently as when we induce it in the neuroectoderm during NB specification (Fig. 6F), further suggesting that the neuroblast program at the chromatin level commences during the NE to NB transition.

The foregoing experiments demonstrated that although individual ASC proneurals are sufficient to rescue the CNS defects caused by *ASC* deletion, none of their other primary targets tested were competent to do so (Fig. 6B–E). However, in the presence of proneural proteins (in wt background), *scrt*, *wor*, and *dpn* neuroectodermal overexpression by bib-Gal4 led to significant neural hyperplasia evident at the level of longitudinal connectives and segmental/ intersegmental nerve bundles (Additional file 1: Fig. S8A). Cuticle preps showed epidermal holes (Additional file 1: Fig. S8B), suggesting that *scrt*, *wor* or *dpn* NE overexpression tipped the balance in favor of NB specification at the expense of epidermis. Although, in the wild-type context, bib>scAPAA overexpression on its own had a weak effect (Additional file 1: Fig. S1B-C), coexpression with *dpn* enhanced the hyperplasia produced by either alone (Additional file 1: Fig. S8A). Similar enhancement was observed upon co-expressing two proneurals together, *scAPAA* with *l(1)sc* (Additional file 1: Fig. S8A). Notably, VNC hyperplasia was not seen when these genes were induced in the neuroblasts by pros-Gal4 (starts expressing in st11 NBs, GMCs and neurons) (Additional file 1: Fig. S8C) or in neurons using elav-Gal4 (starts expressing in st13 NBs, GMCs and neurons, not shown). These results suggest that TFs of the Snail (Wor, Scrt) and Hes families (Dpn), most known to act as repressors [86, 87], can enhance the NB-promoting activity of proneural TFs, but have little genuine activating potency to initiate the neural program on their own (Fig. 6H, I, model cartoons). This conclusion is supported by the ectopic generation of neural cells in the wing disk induced by a TF cocktail consisting of a proneural (Ase), a Snail (Wor), as well as two more broadly NE-expresssed TFs (SoxN and Kr) [88].

### Proneural bound cis-elements exhibit enhancer activity and proneural dependency

**Fig. 7:**
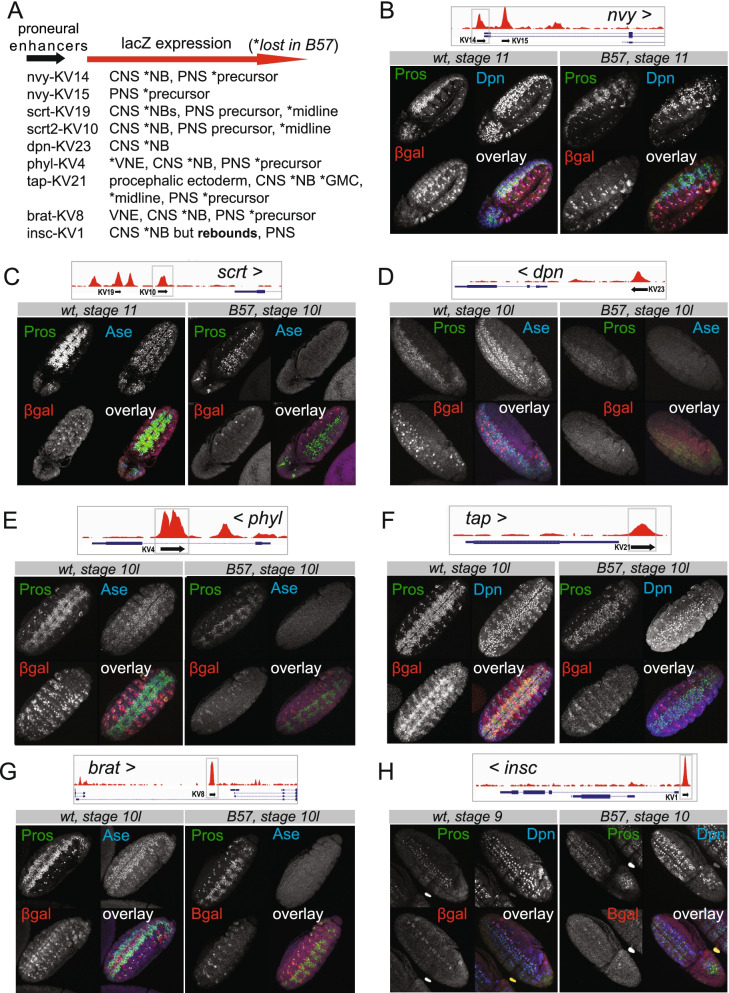
Proneural bound genomic elements exhibit spatiotemporal enhancer activity and proneural dependency. **A** Summary of enhancer spatiotemporal expression patterns in wt and Dfsc(1)scB57 (*) embryos. **B** Embryos expressing the upstream nvy-KV14 reporter. **C** Embryos expressing the upstream scrt-KV10 reporter. **D** Embryos expressing the upstream dpn-KV23. **E** The intronic phyl-KV4 reporterin stage 10 embryos. **F** The 3′ prime tap-KV21 reporter. **G** The KV8 reporter proximal to the short brat isoforms **H** The proximal to TSS insc-KV1 reporter. In the genomic insets, black arrows indicate the extent and cloning orientation of the genomic elements in the lacZ expressing vectors. The > symbol next to gene names shows the orientation of transcription. Overlay is the composite of the three channels. The genomic coordinates and characteristics of proneural bound enhancer regions cloned for the generation of lacz reporter flies provided in Additional file 2: Table S7

To investigate the transcriptional activity of the proneural bound elements we generated 10 transgenic *lacZ* reporter flies. We selected proneural peaks, near *nvy*, *dpn*, *scrt*, *wor*, and *tap* genes, whose protein products showed proneural dependency in mutant embryos in our foregoing analysis. We included binding events near *insc* and *brat*, two key neuroblast genes that are implicated in apico-basal polarity and asymmetric cell division [89], and one intronic peak from the *phyl* gene, a known PNS proneural target [90]. Most of these regions coincided with DHS sites and half had Zelda binding during MZT (Additional file 2: Table S7). All fragments showed enhancer activity in some regions of the developing nervous system, central and/or peripheral, and none in non-neural tissues. The wor-KV29 exhibited weak expression and was not studied further. For the remaining lines, we compared the *lacZ* expression patterns in wildtype and *Df(1)scB57* embryos, summarized in Fig. 7A.

Briefly, the *nvy* enhancers, exhibited different patterns, nvy-KV14 had CNS and PNS expression (Fig. 7B) while nvy-KV15 was PNS exclusive (not shown). In mutant neuroblasts, nvy-KV14 expression was abolished throughout neurogenesis similar to the Nvy protein (Fig. 4C, D). In scratch-KV10-lacz wt embryos, we detected moderate NB and stronger midline signal, which was lost in mutants (Fig. 7C) in contrast to the rebound in scrt-GFP protein (Fig. 4F). dpn-KV23, was expressed in S3 and S4 NB waves and by stage 13 had expanded to cover the whole NB pool (not shown). In the *Df(1)sc-B57* mutant, KV23 was never activated (Fig. 7D), in contrast to the resumed Dpn protein expression (Fig. 4). The phyl-KV4 enhancer expressed from st9/10 in a NB subset and some VNE clusters was lost in the mutant background (Fig. 7E). Next, tap-KV21-lacZ, exhibited ectodermal, CNS (subset of NBs and GMCs) and PNS expression (Fig. 7F). In early mutant embryos, the NB/GMC expression was lost (Fig. 7F) but we did detect limited expression in GMCs and midline from stages 13–14 onwards (not shown). Similarly, brat-KV8-lacZ (Fig. 7G), exhibiting broad neuroblast expression in wt embryos, lost its expression in the mutant background, even after the onset of asymmetric divisions and generation of Pros positive GCM progeny. Lastly, the insc-KV1 enhancer showed extensive NB expression from S1-S2 onwards with an emphasis in the lateral and intermediate rows. It exhibited absence of expression in mutant NBs during the stalling window but did express weakly during the rebounding period (Fig. 7H).

Thus, with the sole exception of the *insc* enhancer, the NB-specific activity of *nvy*, *scrt*, *dpn*, *phyl*, *brat*, and *tap* regulatory elements exhibited absolute ASC dependency both during stalling as well as after stem cell activity resumption. This suggests that, at the chromatin level, the delayed NB activation in the absence of proneurals is mediated by cis-elements distinct from those bound by proneural proteins. Unlike NB expression, all enhancers that drove PNS expression displayed activity in the *Df(1)scB57* mutant in the ASC-independent sensory organs [91], most likely due to the activity of the *atonal* and *amos*, proneural factors exclusive to PNS primordia [92, 93].

## Discussion

### Chromatin dynamics during embryonic nervous system development

By mapping ASH binding events during neural stem cell specification, we found a high co-occurrence with accessible regions pre-modeled during MZT, a time when Zelda is crucial for establishing chromatin organization for subsequent tissue-specific transcription [60, 94]. Since ASH proneurals are among the earliest zygotically transcribed genes [57, 95], we hypothesize that they may survey the early gastrula chromatin to gain access to neurogenesis related enhancers and possibly pre-initiate target transcription. This notion is supported by a single-cell RNA-seq study of the early gastrula where the neuroectoderm primordium cell cluster expressed sc and some of its direct targets as identified here [96]. Later in the mature neuroectoderm, we demonstrate that proneurals also bind Zelda-independent elements, which showed restricted accessibility earlier at the onset of zygotic transcription. ASH binding at these enhancers and concomitant gain in histone activation marks near known neural stem cell genes demonstrates their activating potency.

### ASC proneurals mediate the timely activation of the neural stem cell program in the neuroectoderm

Our work indicates that during NE to NB specification spanning stages 8-11, proneural-mediated chromatin reorganization and transcription is essential for the proper later unfolding of the entire NB lineage. For the first time, we demonstrate that proneurals establish NB homeostasis of all 5 delamination waves, based on our genomic data (Fig. 2B), the phenotypic analysis of mutant NBs, both early (Fig. 4) and late born (Fig. 5) and the expression patterns of the cloned proneural enhancers in vivo (Fig. 7). Thus, as reported for a single neuroblast, the MP2 [83, 84], it appears that all NBs that manage to delaminate in *ASC* mutants are mis-specified and cannot overcome functionally the initial stalling. Interestingly, murine *Ascl1* depleted neural precursors also exhibit a similar delay [97].

Although proneural factors are crucial in the timely execution of the NB transcriptional program, partial activation of the program happens in their absence (Fig. 4). This is most likely mediated by different enhancers than those bound by ASH proteins as shown by the expression of lacz-reports in the *ASC*^*-*^ background (Fig. 7). The elusive proneural factors in ASC null embryos have been a long-standing puzzle [47, 98]. Such TFs could be Hb, in collaboration with Sna [99], since the expression of both was only mildly affected by *ASC* loss (Fig. 4, Additional file 1: Fig. S5). Another possibility would be Daughterless, which heterodimerizes with ASH proteins, but also functions as a homodimer [54, 100]. Earlier observations have shown that L(1)sc and Ase can bind DNA as homodimers in vitro [48]. From the narrow overlap of our proneural binding consensus with Da (Additional file 1: Fig. S1G) it is conceivable that in the embryonic neuroectoderm the two act to a large extent via distinct enhancers, contrary to the current belief that proneural factors are obligate heterodimeric partners of Da. This also agrees with the strong enhancement of the neural hypoplasia of double *ASC* and *da* mutants [47]. On the other hand, it is unlikely that Wor and SoxN are the compensating proneural TFs as proposed by [98]. That study demonstrated that Wor and SoxN use their repressive capacities to promote neurogenesis, since EnR (Engrailed repression domain) fusions phenocopied their effect upon ectopic expression in epithelial cells [88]. It is unlikely that a duo of repressors would be able to activate the large cohort of NB specific genes that seems to be turned on by proneural factors (our study). In fact we have shown that *wor* is under ASH transcriptional control (Fig. 3C, Additional file 1: Fig. S3A) and reinstating its expression in *ASC* mutants is insufficient to rescue the CNS hypoplasia (Fig. 5C), although it mildly improves NB recovery from stalling. Regardless of the identity of other NB-promoting TFs, the eventual initiation of proliferation and rebound in the expression of key identity genes in *ASC* deficient NBs is insufficient to restore neural programming at the organism level, as evidenced by the depleted neuronal/glia progeny. This suggests that the ASC TFs are vital for neural stem cell homeostasis.

Our work favors a model whereby ASC proneurals turn on the NB stem cell identity program, rather than promoting their delamination per se. From a combination of early NB markers (Additional file 1: Fig. S5), we believe that almost all NBs delaminate in the ASC null mutant, more than previously thought, although we cannot rule out the possibility that a few may be missing. We can also not rule out a possible delay in the delamination process, since no single NB marker can consistently mark all mutant NBs. Although wt NBs express both markers robustly by early st 9, mutants show regional delays in Hb accumulation (Fig. 4G, Additional file 1: Fig. S5B) as well as in Sna accumulation (Additional file 1: Fig. S5C). This confirms the identity defect of these cells, even when considering the less severely affected markers Hb and Sna.

### Networks downstream of proneurals

Integration of the proneural binding events with the RNA-seq and H3K27Ac changes during Notch-mediated lateral inhibition revealed a downstream TF network, likely to consolidate the neural cell fate. Some of these had been earlier described as potential proneural target genes, like *dpn* [101], *scrt* [102], *ase* [48, 49], and *nerfin1* [103, 104]. On the other hand, *sna* had been reported not to be a proneural target [105] despite several proneural binding events in its vicinity (Additional file 2: Tables S1 and S6). Some of these genes had also been reported to be repressed by Notch signaling, notably *scrt* [106], *sna*, *dpn*, and *ase* (same refs as above). It is known that the transition from neuroectoderm to NB involves a mutually antagonistic interplay between the proneurals and Notch; however it remains unclear to date if the effect of Notch on the proneural targets is indirect, via Notch repressing ASC proneural expression, or direct on their individual enhancers, which are activated by ASC proneurals. The dominant effect of Notch activation even upon overexpression of a proneural factor [107] argues in favor of a direct effect of Notch on proneural targets, although a combination of direct and indirect action is conceivable.

How do these NB-specific TFs ensure the establishment of the NB fate? Ase plays a central part in this TF network as being the only NB-specific TF with a potent activating function [108]. The overlap of NE-expressed ASH binding events with NB-expressed Ase binding (Additional file 1: Fig. S1F) suggests that in the neuroectoderm ASH proneurals may mark neural enhancers which Ase will subsequently sustain to unfold the NB program. This is demonstrated in the sc19 deficiency where the presence of Ase partially improves mutant NB functionality and progeny development, compared to the deletion of all four ASC members (Additional file 1: Fig. S7). However, we find it impressive that the neuroectodermal ectopic induction of Ase can almost fully rescue the neurogenesis defects caused by *ASC* deletion (Fig. 6F), proving, first, its functional equivalence to ASH TFs and, second, that the neural program is activated early on during neural stem cell selection.

The remaining TFs of this network are in their vast majority transcriptional repressors, highlighting the importance of blocking alternative transcriptional programs and differentiation fates to ensure the proper unfolding of the NB program. We show that single members of this network contribute to neurogenesis, but we believe they mainly work combinatorically and in parallel to an ASC factor [88]. Snail TFs are central in this network and appear to have pivotal roles in NS development [73, 99, 109]. Snails however are not essential for NB ingression [70], instead, it seems that they regulate NB function and GMC transition [99, 110]. In addition to these core downstream TFs, NE proneurals bind near >1000 genes, which may contain previously uncharacterized players in implementing the NB fate and launching the subsequent GMC and neuron/glia developmental programs.

### Proneurals pioneer differentiation programs partly in the stem/progenitor cell

The mature VNC pattern is the outcome of a complex crosstalk of glia and neuron interactions occurring in the CNS, midline [78] and PNS [79]. Our identified proneural binding events near genes of all nervous sub-systems validate the genetic evidence of ASC involvement in their development [42, 47, 91, 111]. We thus propose that the late CNS defect in *ASC* embryos is the collective outcome of impaired stem cell specification and impaired progeny from different sub-systems, failing to establish the necessary communication cues. For example, the absence (or mis-specification) of longitudinal glia (defective GP lineage) and the dMP2/vMP2 segment-spanning interneurons (defective MP2 lineage) could be the underlying cause for the lack of longitudinal tracts that eventually may lead to nerve cord fragmentation.

In addition, studies in flies and mice have shown that, besides stemness, proneurals impact neuronal differentiation as well [112–116]. In our work, we identified binding near genes expressed in later differentiated cell types, GMC, neurons, and glia (Fig. 2), where *ASC* gene expression has been extinguished. For at least one of these genes, *tap*, we showed that its protein expression is greatly compromised in *ASC* mutant GMCs (Additional file 1: Fig. S4C). We envision that this is happening in two ways: First, proneurals could regulate chromatin dynamics at neuronal/glial enhancers during neuroblast specification but robust transcriptional activation only happens later, delegated to TFs that appear as the neural differentiation program unfolds. Indeed, comparisons of chromatin states between stem cells and neurons support this notion. Some CNS-specific enhancers are “constitutive,” i.e., accessible from the NB all the way to neurons, whereas other neuron-specific enhancers gradually become accessible at later embryonic stages [5]. A second, not mutually exclusive, scenario is that key neuronal transcripts produced at the NB stage, are translationally repressed. Such genes are most likely pro-differentiation factors that generally lock cellular identity, as has been shown for the *elav* gene, whose transcription initiates in many cell types, but its protein product is strictly neuron-specific [117].

## Conclusions

We demonstrate that during neural stem cell specification ASC proneural TFs modulate chromatin dynamics to achieve the timely activation of neural transcription, promoting stemness but at the same time paving the way for appropriate lineage differentiation. Importantly, the action of proneurals on chromatin has to take place early on, as NBs delaminate from the neuroectoderm. We envision that all stem cells and their future lineages within a tissue may depend on similar mechanisms of early chromatin remodeling, which is necessary for correct subsequent differentiation events.

## Methods

### Drosophila stocks

UAS-CD8-GFP (II); bib-Gal4 (III) homozygous females were crossed to homozygous UAS-6xmyc-scAPAA, UAS-6xmyc-l(1)sc or UAS-NΔecd males for the embryo collections used in ChIP-seq and RNA-seq experiments. The Df(1)sc-B57 and Df(1)sc^19^ flies where rebalanced with a FM7, KrGal4, UAS-GFP chromosome to enable distinguishing the mutant embryos during imaging. Df(1)scB7/FM7,KrGal4,UAS-GFP(I); bib-Gal4(III) females were used for the UAS rescue experiments and for the UAS-FUCCI experiment.

For the generation of UAS-l(1)sc N-terminally 6xmyc-tagged flies, the l(1)sc coding region was amplified using primers with EcoR1 XhoI restriction sites overhangs (EcoR1-forward, XhoI-reverse) from yw cDNA (Superscript III, ThermoFisher 18080093), using KAPA High Fidelity Polymerase (Kapa/Roche, KK2103) and subsequently inserted in the entry pENTR™3C vector (ThermoFisher, A10464). We used pTMW (Drosophila Genomics Resource Center #1107) as the destination vector and the Gateway® LR Clonase® II kit (ThermoFisher, 11791020) to generate the final l(1)sc-pTMW vector. Subsequently the l(1)sc-pTMW construct was inserted into yw flies via P-element transformation. For the generation of enhancer-lacZ reporter flies we used the pBlueRabbit lacZ vector, which contains an hsp70 minimal promoter upstream of a lacZ reporter gene (Housden et al. 2012). Putative proneural bound regions were amplified with the corresponding primers with overhangs for EagI (forward primers) and XbaI (reverse primers) (see Table S7) from Oregon-R genomic DNA extracted with DNAzol™ (Theromofisher). PCR fragments were extracted from agarose gels (Macherey-Nagel, 740609.250). pBlueRabbit vector was digested with EagI and XbaI, gel extracted and dephosphorylated prior to ligations. Constructs were transformed using the φC31 integrase system into y w nos-int ; attP40[y+] / (CyO) hosts. All vectors generated for fly transgenesis were Sanger-sequence verified (Macrogen Inc). A complete list of fly strains and primer sequences are in Additional file 3: Supplemental Methods.

### Embryo collections, immunostaining, and imaging

Embryo collections were made on cherry juice agar plates. Embryos were dechorionated in 50% bleach for 2 minutes. Dechorionated embryos were transferred to 4-ml glass tubes containing fixative solution (1200 μl 1× PBS, 800 μl 10% formaldehyde, 2 ml heptane) and fixed for 20 min with vigorous agitation. Embryos were devitellinized by vigorous shaking in methanol for 30–40 s. After 3 quick methanol rinses, samples were stored in methanol at -20 °C. On the day of immunostaining, embryos were rehydrated in PT (1xPBS, 0.2% Triton). Blocking was then conducted for at least 2 h with PBT (PT+ 0.5% BSA). Primary antibodies were diluted in PBT and incubated overnight at 4 °C. The next day, samples were washed extensively in PT. Embryos were incubated with secondary antibodies for 3 h at room temperature. After extensive PT washes, 80 μl n-propyl gallate-glycerol mountant was added to each sample and incubated overnight at 4 °C. Embryos were then mounted and imaged in TCS SP8 confocal microscope system (Leica). Image analysis was performed with the Leica LAS X software. Antibodies used are listed in Additional file 3: Supplemental Methods.

### ChIP-seq protocol for low embryo number

We developed a low-input Drosophila embryo ChIP-seq protocol based on [118]. Briefly, we set cages of 150 homozygous UAS-CD8-GFP (II); bib-Gal4 (III) female flies with 50 males homozygous for either UAS-scAPAA (II) or UAS-l(1)sc (II), or UAS-NΔecd (II), pre-conditioned for two days in vials before transfer to the cages. All embryo collections were performed during the same time window, from morning to mid-afternoon, to minimize clock-mediated changes in gene expression. A 30-min preclearing step was performed every morning of collection. Egg lays were done on cherry juice/agar 6cm dishes for 0–3 h at 27 °C followed by a 3-h maturation step at 29 °C to boost GAL4 activity. We collected 3–6 hs embryos on a Nitex mesh, dechorionated with 50% bleach for 2 min and washed with water. Subsequently, embryos were transferred with a brush in fixing solution and shaken for 10′ mildly in 2 ml ependorfs. Fixing solution: 1500 μl Heptane, 100 ul 10% FA, 200 μl 10 X PBS and 200 double-distilled H2O. Next, FA was quenched with glycine for 5′ min with mild shaking. Fixing solution was discarded and embryos were washed twice with cold 1× PBS/0.1% Triton-X and then briefly low-speed centrifuged to pellet embryos. After discarding the second PBS wash, embryo pellets were stored in – 80 °C. A detailed protocol can be found in Additional file 3: Supplemental Methods.

### Drosophila embryo RNA-seq

Embryos were collected at 0–2hs and then transferred to mature at 29 °C for 3 h (3-5hs collections). All embryo collections were performed during the same time window, from morning to mid-afternoon, to minimize clock-mediated changes in gene expression, after a 30-min pre-clearing. Embryos were directly transferred in 50 μl TRIzol-containing tubes and stored at − 80. On the day of RNA extraction, embryos were defrosted and homogenized using 1.5 ml manual pestle. For each replicate, 5 independent daily collections were pooled after homogenization and RNA was isolated with phenol/chloroform without columns. RNA-seq libraries construction was performed with the Ion Total RNA-seq Kit v2 (Thermo Fisher), using Poly(A) RNA selection with Dynabeads mRNA DIRECT Micro Kit Ambion (Life Technologies) according to manufacturers’ protocols. Libraries were sequenced on Ion Proton™ System (ThermoFisher) with PI CHIP v3, utilizing for template the Ion PI Hi-Q OT2 200 kit (# A26434) and the Ion PI Hi-Q Sequencing 200 kit (# A26433, A26772).

### NGS data analyses

Fastq files were transferred from Ion Proton and Illumina Basespace to IMBB servers for storage and analysis. Mapping was performed to dm6 (UCSC/dm6, iGenomes, 2015). Software and Algorithms used in this study: SAMtools [119], MACS2 (v1.4) [120], HOMER (v4.5) [121], Hisat2 [122], Cutadapt (v1.12) (doi:10.14806/ej.17.1.200), HTSeq [123], edgeR [124], BEDTools [125], deepTools [126], GSEA (v4.0.3) [127], R (v4.0.3) (https://www.R-project.org/), Pavis (https://manticore.niehs.nih.gov/pavis2/ Flybase R6.01 assembly) [128], Flymine (https://www.flymine.org v51) [66], i-cis Target https://gbiomed.kuleuven.be [129], UCSC genome browser [130] (FlyBase/BDGP/Celera Genomics Release 6 + ISO1 MT), Flybase [67] http://flybase.org/cgi-bin/cvreport.pl?cvterm=FBbt:00001369&childdepth=2, FBbt:00001369 (Fly Anatomy) “VNC neuroblast” genes used to generate Fig. 2A, BDGP in situ https://insitu.fruitfly.org downloads/insitu_annot.csv.gz was used to run the GSEA presented in Fig. 3F, G.

### ChIP-seq peak calling, motif analysis, and genomic annotation

Mapping was performed using Hisat2 (--no-spliced-alignment --score-min L,0,-0.5), (samtools view -q 30). Bedgraphs were generated using bedtools genomecov and uploaded to the UCSC genome browser. Prior to peak calling, we excluded reads from the bam files mapped on repetitive regions. We also excluded reads that fell in our custom “black list regions” (available upon request). Peak calling was performed using macs2 over input (-p 0.05) and peak overlaps were generated with bedtools (intersect -wa), excluding Chromosomes U and Uextra. This resulted in 4129 common peaks between the two biological replicates. In order to derive a more confident proneural consensus, we used the second scAPAA, rep which was stronger, and imposing an FC>2 filter, based on the enrichment score from the macs2 output file of the 2nd rep. This resulted in 2,984 confident proneural consensus peaks, commonly called in both replicates (Fig. 1D). Motif analysis was done with homer findMotifsGenome.pl –size given. Assignment of peaks to genes was performed using homer annotatePeaks.pl. The genomic distribution of the datasets was performed by homer annotatePeaks.pl dm6 (default) and Pavis with parameters of upstream and downstream length set at 5 kb.

### Proneural peak consensus overlapping with Zelda and chromatin marks during MZT

We overlapped our proneural binding consensus with Zelda binding events during blastoderm cellularization (the time of the maternal to zygotic transition) from two studies [57, 59] and found 41% and 62% overlap respectively. The proneural.vs.Zelda.Harrison data overlap was a subset of the proneural.vs.Zelda.Sun; therefore, we decided to continue with the second, presented in Fig. 1, since it gave higher overlap with the proneural cistrome. We used Table S5 from the Harrison study and the GSE65441_Zld_DESeq.txt.gz from the Sun study. Both datasets were converted to Drosophila genome version dm6 from dm3 using LiftOver in the UCSC browser.

### Proneural consensus overlaps with modENCODE datasets

For the DHS st5-st14 dataset [8], we downloaded the bed files of coordinates of 5% FDR peaks from the UCSC genome browser https://genome.ucsc.edu/index.html and regulation/track:BDTNP DNase Accs/table:S5-14 Regions (bdtnpDnaseAccS5-14) and then used UCSC/LiftOver to convert to dm6. ChIP-seq data for Ac (ENCFF073ETO), Da (ENCFF718YZD), E(spl)m8 (ENCFF074INK) were downloaded from https://epic.gs.washington.edu/modERN/.

### Heatmaps of ChIP-seq datasets

We downloaded and mapped to dm6 parameters from the following Illumina sequencing datasets: SRR1779551 (Zelda) and its input SRR1779552. NC14 histone marks SRR1505729 (H3K27me3), SRR1505714 (H3K27Ac), SRR1505718 (H3K4me1), and SRR1505740 (input). SRR388356 (PolII) and SRR388382 (input). To correct for the difference in fragment size between Ion Torrent and Illumina (75bp) sequencing we processed the IonTorrent datasets as follows: fastq reads were filtered and trimmed using cutadapt -m100 -l100 prior to Hisat2 mapping (--no-spliced-alignment --score-min L,0,-0.4 and samtools view -q 30). We indexed all bam files and used deepTools bamCompare, computeMatrix, plotProfile for Fig. 1H and plotHeatmap to generate Figs. 1G and 3B. We used as reference regions the center of proneural binding events (class I and II) ±5 kb from peak center. Heatmaps in Figs. 1D and S1E were generated from the mapped reads, unprocessed for length, normalized over input, using ±5kb from proneural peak centers, using a custom script from the Odom lab [131], exported to images by TreeView software from the Eisen lab.

### Boxplot of ChIP-seq datasets

For the boxplots in Fig. 3D and Additional file 1: Fig. S2E, we generated the RPKM counts for each H3K27Ac library from bam files (excluding black list and repeat regions in advance). For the Torrent libraries (rep1), we used the processed and trimmed to 100bp reads to be more comparable with the 75bp Illumina reads (rep2). We then used the deepTools bamCoverage –normalizeUsing RPKM to generate bigwig (bw) files for each library. Subsequently, bw files were used to generate the RPKM count matrices using deepTolls/multiBigwigSummary on genomic regions specified. Boxplots were generated in R (4.0.3) using the log2 of the RPKM input counts. Statistics were performed with Wilcoxon rank-sum tests.

### Differential acetylation analysis of DHS sites

We generated the RPKM count matrix with multiBigwigSummary on the total of 16,512 st. 9 DHS sites [8] with the RPKM.bw files for each H3K27Ac library. Subsequently, we imported the combined RPKM.count file as a DGEList in R/edgeR. Filtered out DHSs with cpm cutoff of 20 resulted in 14,334 DHS regions. Model.matrix was generated using (~0 + group + batch). The mean-variance relationship of the log-RPKM counts was estimated by limma/voom. We used a linear model (lmFit) and Empirical Bayes statistics (eBayes) in two-group contrasts to assess differential H3K27Ac occupancy. Output of the analysis is provided in Additional file 2: Table S4.

### RNA-seq differential analysis

Mapping was performed using Hisat2 (ref, --score-min L,0,-0.5). Counts were generated from bam files with HTSeq-count (-i gene_id). Differential Expression Analysis was performed with edgeR using batch correction and likelihood ratio tests (glmFit/glmLRT method), since replicates were performed in different time points resulting in large dispersions within groups. Tests were performed on 7862 genes after keeping genes with cpm>3 in at least 3 samples. edgeR output of the U-scAPAA vs. U-NΔE analysis is provided in Additional file 2: Table S5. GSEA (https://www.gsea-msigdb.org/gsea/index.jsp) was performed using the ranked gene lists from the edgeR output files against the BDGP in situ database terms and genes assigned to proneural peaks or affected DHS sites.

## Supplementary Information

**Additional file 1: Fig. S1.** Embryonic phenotypes of bib-Gal4 driven expression of U-scAPAA and U-NΔE and genomic analyses of proneural binding consensus. **Fig. S2.** Proneural regulated chromatin effects correlate with transcriptional output. **Fig. S3.** ASC mutant neuroblasts are initially arrested at G2/M. **Fig. S4.** GMC expression of proneural targets is impaired in ASC mutants. **Fig. S5.** A study of several neuroblast markers reveals the presence of delaminated ASC mutant NBs. **Fig. S6.** Depletion of glia and neuronal populations in ASC mutants result in severe axonal defects. **Fig. S7.** Asense provides partial neuroblast functionality and CNS development in the absence of ac, sc and l(1)sc. **Fig. S8.** Neuroectodermal induction of proneural targets enhances neurogenesis at the expense of epidermal fate.

**Additional file 2: Table S1.** Proneural consensus binding sites genomic coordinates and gene annotation. **Table S2.** Motif analysis and genomic distribution of the Class I and Class II Proneural binding events. **Table S3.** Flymine Analysis of Proneural Target Genes. **Table S4.** Differential acetylation analysis of H3K27Ac ChIP-seq signal on stage 9 DHS regions. **Table S5.** Analysis from U-scAPAA versus U-NΔE embryo RNA-seq profiling. **Table S6.** Genomic landscape of selected proneural TF targets. **Table S7.** Genomic characteristics of peaks cloned in lacZ reporter flies.

**Additional file 3: Supplemental Methods.** Fly strains, primers, antibodies, ChIP-seq protocol for low Drosophila embryo number, Ion Torrent Sequencing and Illumina sequencing.

## Data Availability

All data generated or analyzed during this study are included in this published article, its supplementary information files, and publicly available repositories. The raw sequencing data generated in this study are available from the NCBI BioProject database (https://www.ncbi.nlm.nih.gov/bioproject/) under accession number PRJNA719934. Metadata and analyses outputs are available in Additional file 2: Tables S1-S7.xls. *Drosophila* strains generated in this study are available upon request.

## Abbreviations

*ASC*: Achaete-scute complex genes: *achaete (ac)*, *scute (sc)*, *lethal of scute (l(1)sc)*, and *asense (ase)*
*ASH*: *Achaete (ac)*, *scute (sc)*, *lethal of scute (l(1)sc)* genes
cpm: Counts per million
*B57*: *Df(1)sc-B57* mutant fly stain lacking *ac*, *sc*, *l(1)sc*, and *ase* proneural genes
BDGP: Berkeley *Drosophila* Genome Project
ChIP: Chromatin immunoprecipitation
CNS: Central nervous system
DHS: DNase I hypersensitive site
FDR: False discovery rate
GSEA: Gene Set Enrichment Analysis
MZT: Maternal to zygotic transition
NB: neuroblast
RPKM: Reads per kilobase, per million mapped reads
*sc19*: *Df(1)sc*^*19*^ mutant fly stain lacking *ac*, *sc*, and *l(1)sc* proneural genes
TSS: Transcription start site
UTR: Untranslated region
