# Supplementary material for: ASC proneural factors are necessary for chromatin remodeling during neuroectodermal to neuroblast fate transition to ensure the timely initiation of the neural stem cell program
Source: BMC Biol. 2022 May 13;20:107. doi: 10.1186/s12915-022-01300-8 (PMC9102361; doi:10.1186/s12915-022-01300-8)
Supplement: Supplementary file 1 — Additional file 1: Fig. S1. Embryonic phenotypes of bib-Gal4 driven expression of U-scAPAA and U-NΔE and genomic analyses of proneural binding consensus. Fig. S2. Proneural regulated chromatin effects correlate with transcriptional output. Fig. S3. ASC mutant neuroblasts are initially arrested at G2/M. Fig. S4. GMC expression of proneural targets is impaired in ASC mutants. Fig. S5. A study of several neuroblast markers reveals the presence of delaminated ASC mutant NBs. Fig. S6. Depletion of glia and neuronal populations in ASC mutants result in severe axonal defects. Fig. S7. Asense provides partial neuroblast functionality and CNS development in the absence of ac, sc and l(1)sc. Fig. S8. Neuroectodermal induction of proneural targets enhances neurogenesis at the expense of epidermal fate. [file 12915_2022_1300_MOESM1_ESM.docx]

**Additional file 1: Figures S1-S8**


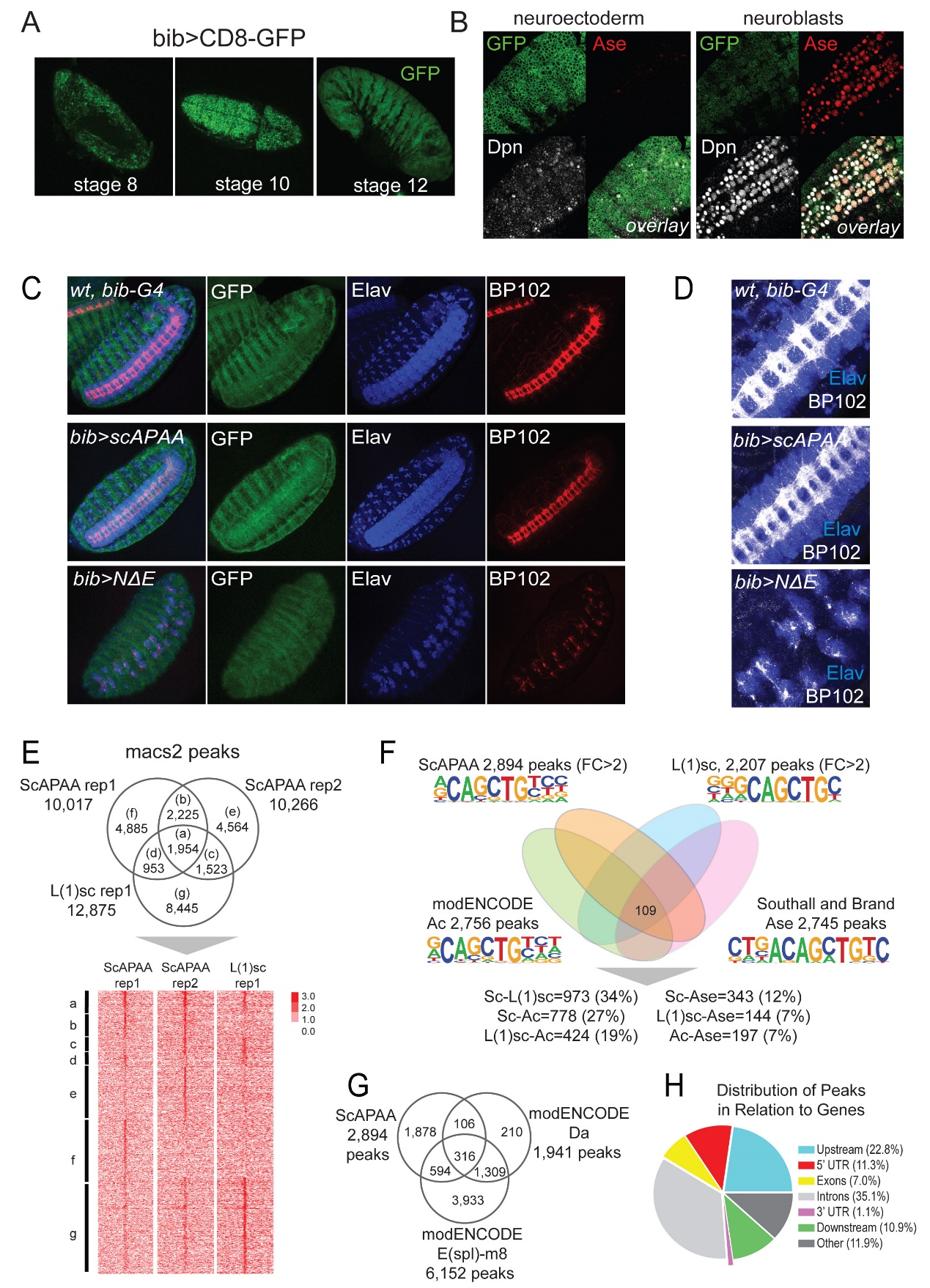


**Fig. S1. Phenotypes of bib-Gal4 driven expression of U-scAPAA and U-NΔE and genomic analyses of proneural binding consensus.** A) Stage 8, 10 and 12 embryos of bib-GAL4 x UAS-CD8-GFP show neuroectodermal expression, which expands laterally at late stages (st 12). B) Stage 10 neuroblasts in bib-GAL4 x UAS-CD8-GFP express residual GFP. Single sections, using the same laser settings, for the neuroectoderm and neuroblast focal planes of the same embryo. Dpn (grey) and Ase (red) are NB markers. "Overlay" shows the superimposition of all three channels. C) Stage 16 embryos showing mild nerve cord hyperplasia in UAS-scAPAA and severe hypoplastic phenotype in UAS-NΔE; Elav (red) marks neuronal nuclei; BP102 (blue) marks axons. D) Neuromeres of stage 16 embryos of UAS-NΔE embryos exhibit clumps of neurons with limited axonogenesis compared to wt or UAS-scAPAA. E) Peak overlaps among the 3 replicates of ScAPAA and L(1)sc ChIP experiments. Heatmap below the Venn diagram shows the corresponding normalized over input signals of read density. F) Overlap at the level of peaks between all 4 ASC family members and the percentages of pairwise comparisons – percentages state the overlap of binding events for the first factor with respect to the second. De novo motif analysis (homer) revealed slight variations in the enriched E-box bHLH motif. G) Venn diagram of binding peaks overlaps among the proneural ScAPAA consensus and Da or E(spl)m8 from modENCODE data. H) Genomic distribution of the 2,984 proneural binding consensus peaks (Pavis). Upstream is 5kb upstream regions from Transcript Start Site (TSS) and Downstream is 5kb downstream regions of Transcription Termination Site (TTS).


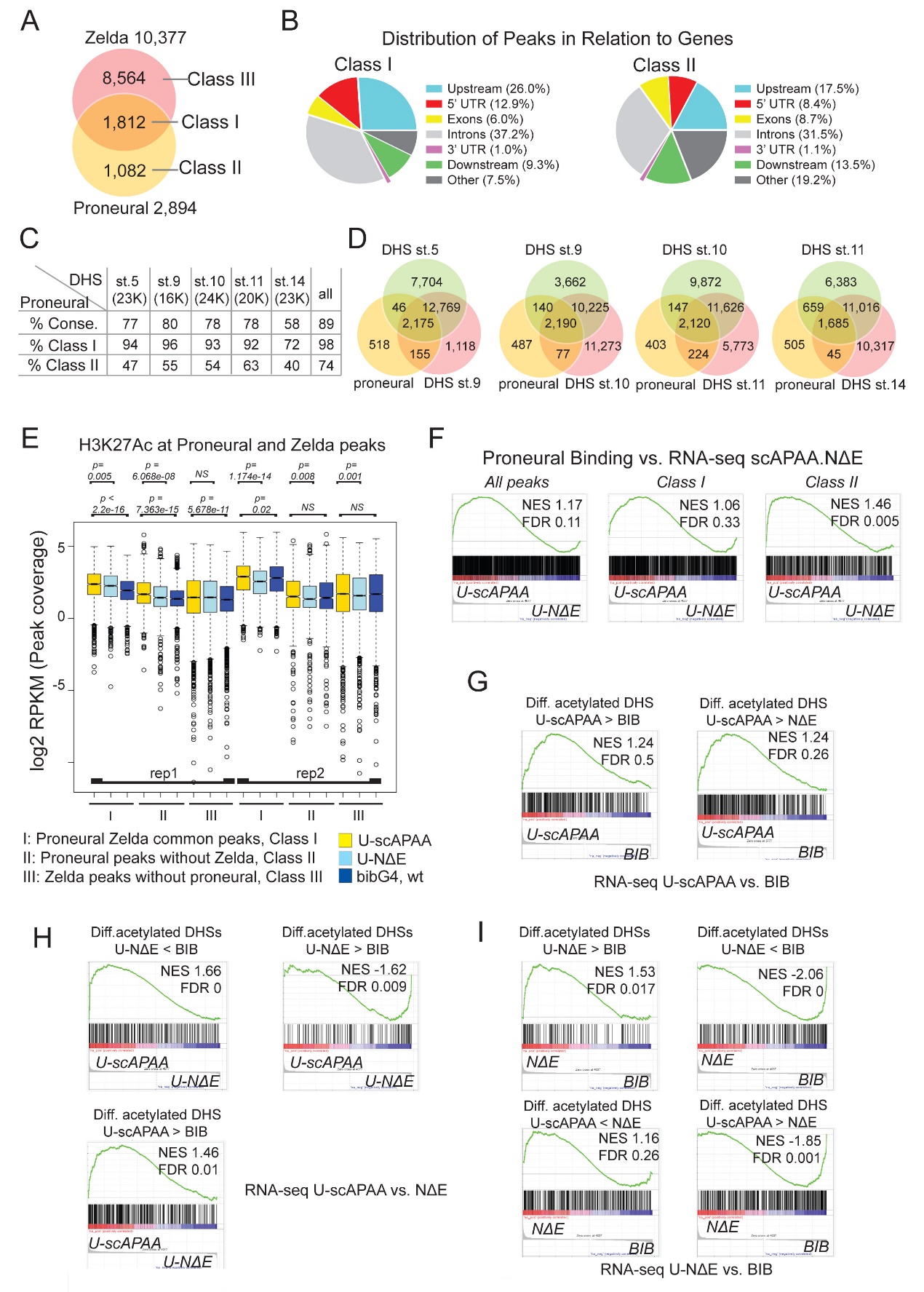


**Fig. S2. Proneural regulated chromatin effects correlate with transcriptional output.**

A) Overlap of proneural consensus peaks during neuroblast specification with Zelda binding events during MZT. B) Genomic distribution of class I and II proneural binding sites (Pavis). Upstream is 5kb upstream regions from Transcript Start Site (TSS) and Downstream is 5kb downstream regions of Transcription Termination Site (TTS). C) Percentage of proneural binding peaks overlapping with stage specific DHS sites from Thomas et al (2011). The top row refers to all 2,894 peaks; the next two rows present Class I and Class II peaks separately. D) Venn diagrams of proneural peaks with consecutive stage specific DHS sites. E) Boxplot of log2 RPKM normalized values of the H3K27Ac ChIP-seq from UAS-scAPAA, UAS-NΔE and wt, bibGal4 embryos on three classes of genomic regions: Class I, 1,812 proneural peaks bound by Zelda during MZT, Class II, 1,082 proneural peaks not bound by Zelda during MZT and Class III, Zelda 8,618 binding peaks during MZT without proneural binding. Statistics were performed with Wilcoxon rank sum tests. F) Gene set enrichment analysis (GSEA) of genes near Class I, Class II or the complete set of proneural consensus peaks with the RNA-seq ranked genes by fold change in UAS-scAPAA vs. UAS-NΔE comparison. G-I) Gene set enrichment analysis (GSEA) plots of the genes near Differentially acetylated DHS sites and the ranked genes from the RNA-seq datasets. G) GSEA plots of genes near Diff.acetylated DHSs with the ranked genes in UAS-scAPAA vs. BIB RNA-seq. H) GSEA plots of genes near Diff.acetylated DHSs with the ranked genes in UAS-scAPAA vs. U-NΔE RNA-seq. I) GSEA plots of genes near Diff.acetylated DHSs with the ranked genes in UAS-NΔE vs. BIB RNA-seq.


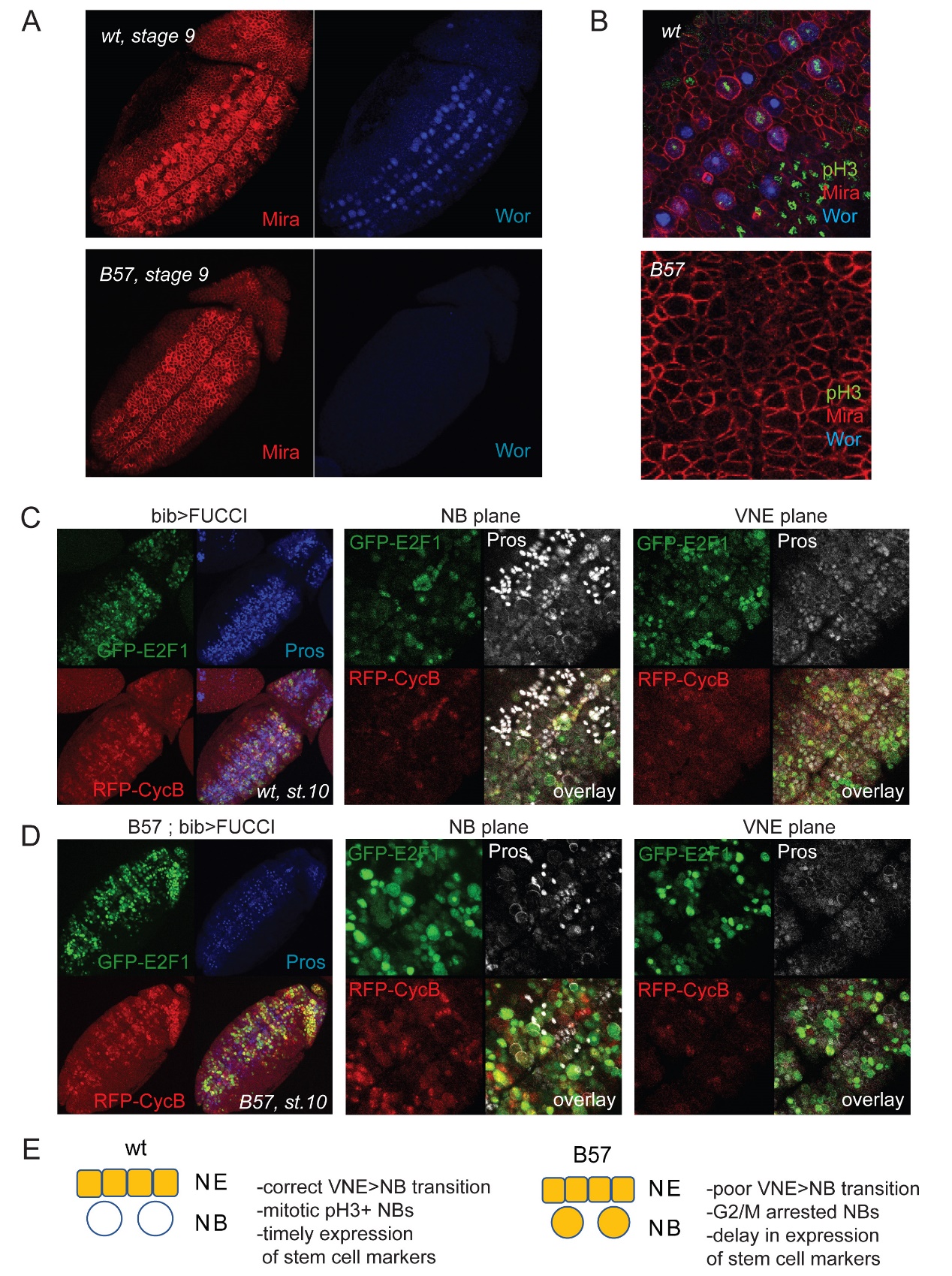


**Fig. S3. ASC mutant neuroblasts are initially arrested at G2/M.**

Stage 9 S1 wt neuroblasts express Worniu robustly, whereas mutants do not. Mira stains the entire ventral NE. B) Single sections at the neuroblast level below the embryo surface shows that S1 delaminated neuroblasts of Df(1)scB7 embryos do not express Worniu nor do they proliferate (pH3), unlike wt neuroblasts. C) bibGal4 wt stage 10 embryo expressing a dual UAS-GFP-E2F1;UAS-RFP-CycB (FUCCI). Left panel shows a projection of a ventral view of the entire embryo, where GFP and RFP are expressed in the neuroectoderm in response to bibGal4. At the NB level (middle panel) limited GFP/RFP expression is detected. Right panel shows strong signal of GFP (G1 marker) at the overlying ventral neuroectoderm level. D) In Df(1)scB7 stage 10 embryo (left) which is just rebounding (few Pros+ GMCs present), many delaminated neuroblasts (middle) that have not divided yet co-express both GFP and RFP suggesting a G2/M arrest, whereas the VNE plane (right) is comparable to wt. In C & D "overlay" refers to the superimposition of all three channels, GFP, RFP and Pros. E) A model cartoon summarizing the phenotype of the delaminated cells in the deletion of ASC proneural genes.


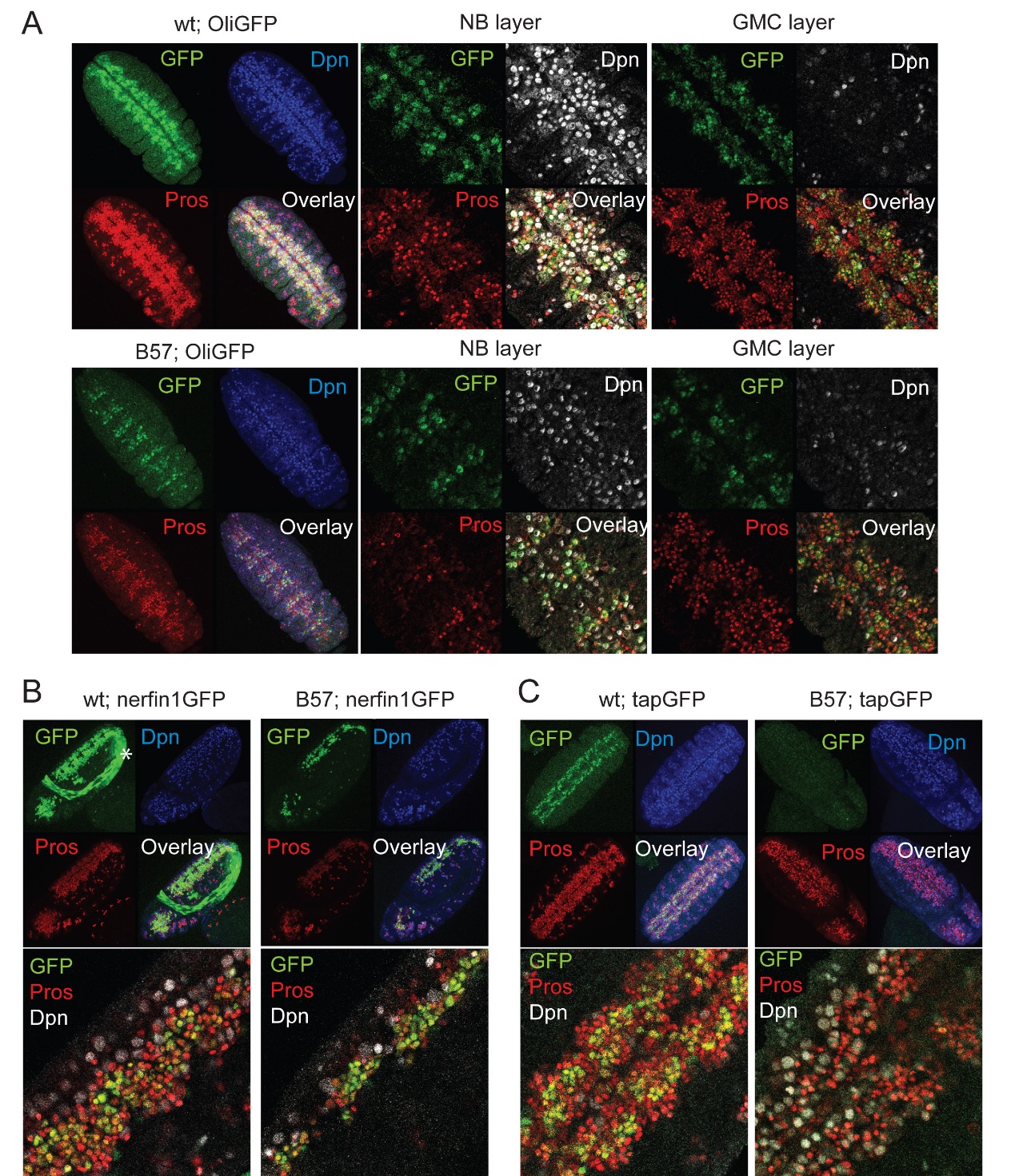


**Fig. S4. GMC expression of proneural targets is impaired in ASC mutants.**

A) OliGFP exhibits neuroblast and GMC expression in wt embryos. In Dfsc(1)B57 after the stalling window OliGFP expression is restricted. B) nerfin1GFP is predominantly expressed in the GMC pool and shows restricted expression in Dfsc(1)B57 embryos. The GFP in the amnioserosa (marked with *) is coming from the FM7, KrGAL4,UASGFP chromosome used to distinguish wt from mutant embryos. C) tapGFP is normally expressed in many GMCs and in Dfsc(1)B57 embryos its expression is not observed during stage 11. In all panels "overlay" refers to the superimposition of all three channels, GFP, Dpn and Pros.


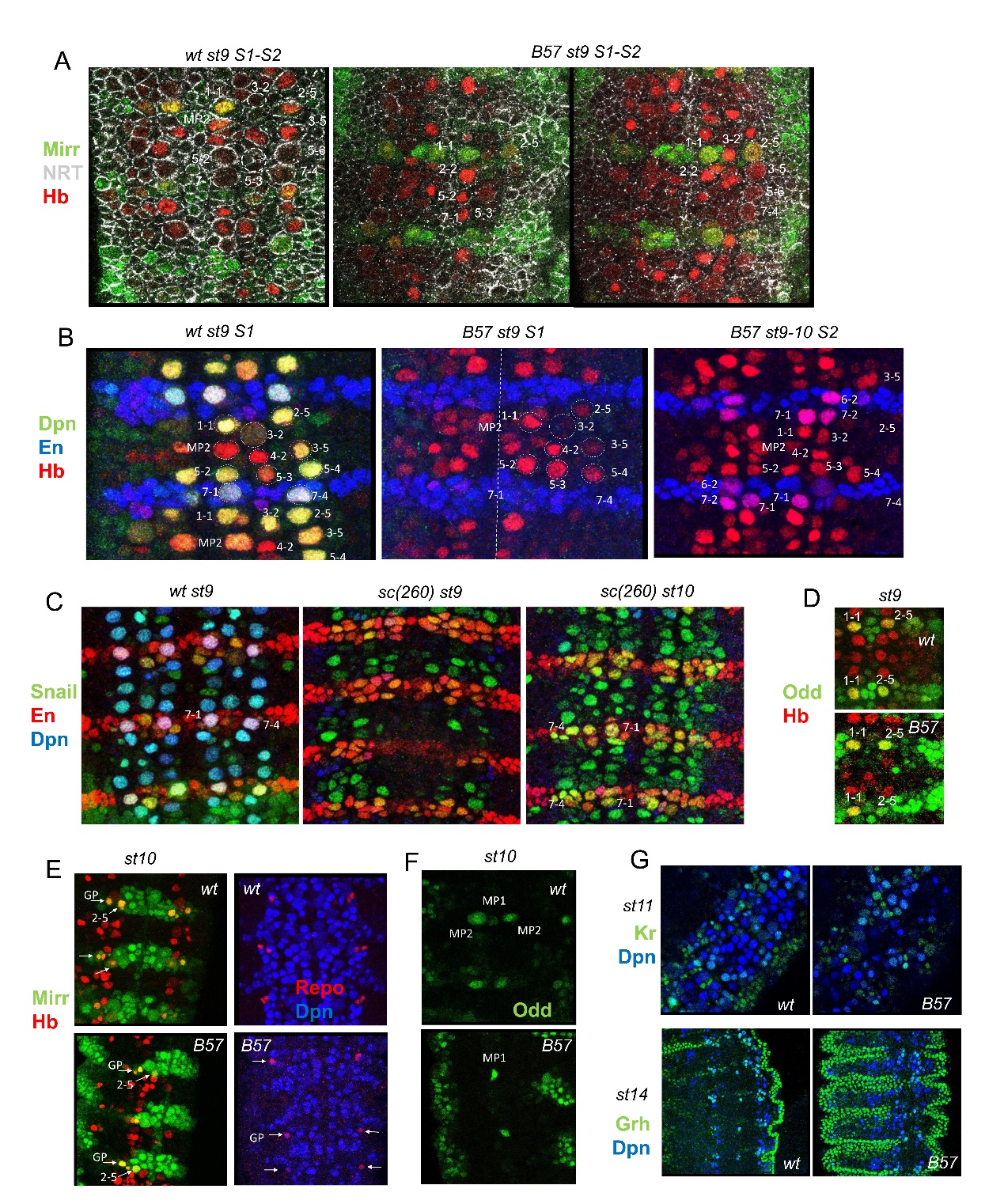


**Fig.S5 A study of several neuroblast markers reveals the presence of delaminated ASC mutant NBs.** A) Stage 9 embryos at the beginning of S2 imaged with Mirr, NRT and Hb. In the wt, NB3-2 and NB5-3 are at a different level above this plane and are shown by dotted outlines. In the B57 embryo (two different focal planes of the same embryo are shown) the10 S1 NBs can be identified. Of note the MP2, not lettered-in, is under the delaminating 2-2. B) Dpn, En, Hb staining reveals that most NBs are present in the B57 st.9. The En+ NB7-1 and 7-4 marks in the middle panel show the assumed position of these neuroblasts, which due to Hb negativity cannot be identified. Note in the later stage 9/10 the appearance of Hb/En NBs. C) wt and Df(1)260.1;snailGFP embryos stained with GFP, En and Dpn. Sna staining reveals the presumptive presence of NB7-1 and 7-4 in the mutant st.10, amongst other neuroblasts. At st 9, Sna expression is still incomplete in the mutant at the NB level shown. D) B57 mutant NB1-1 and 2-5 express Odd-GFP robustly. E) Mirror-GFP in st.10 marks the most dorsally positioned GP and in close proximity to NB2-5. Mutant GPs express Repo in most neuromeres in st.10/11 mutant embryos after the NB Dpn rebound. F) Odd-GFP in wt st.10 embryos marks the midline precursor MP1 and the MP2. In mutant B57 st.10 only the MP1 can be seen by Odd positivity. G) St.11 embryos have progressed in the temporal cascade and NBs start losing Kr expression. In the B57 mutant, st.11 NBs also start phasing out Kr albeit with a delay. In st. 14 many wt and B57 NBs express grainyhead (Grh), suggesting that they completed the temporal series of TF expression. Grh is also strongly expressed in the epidermis.


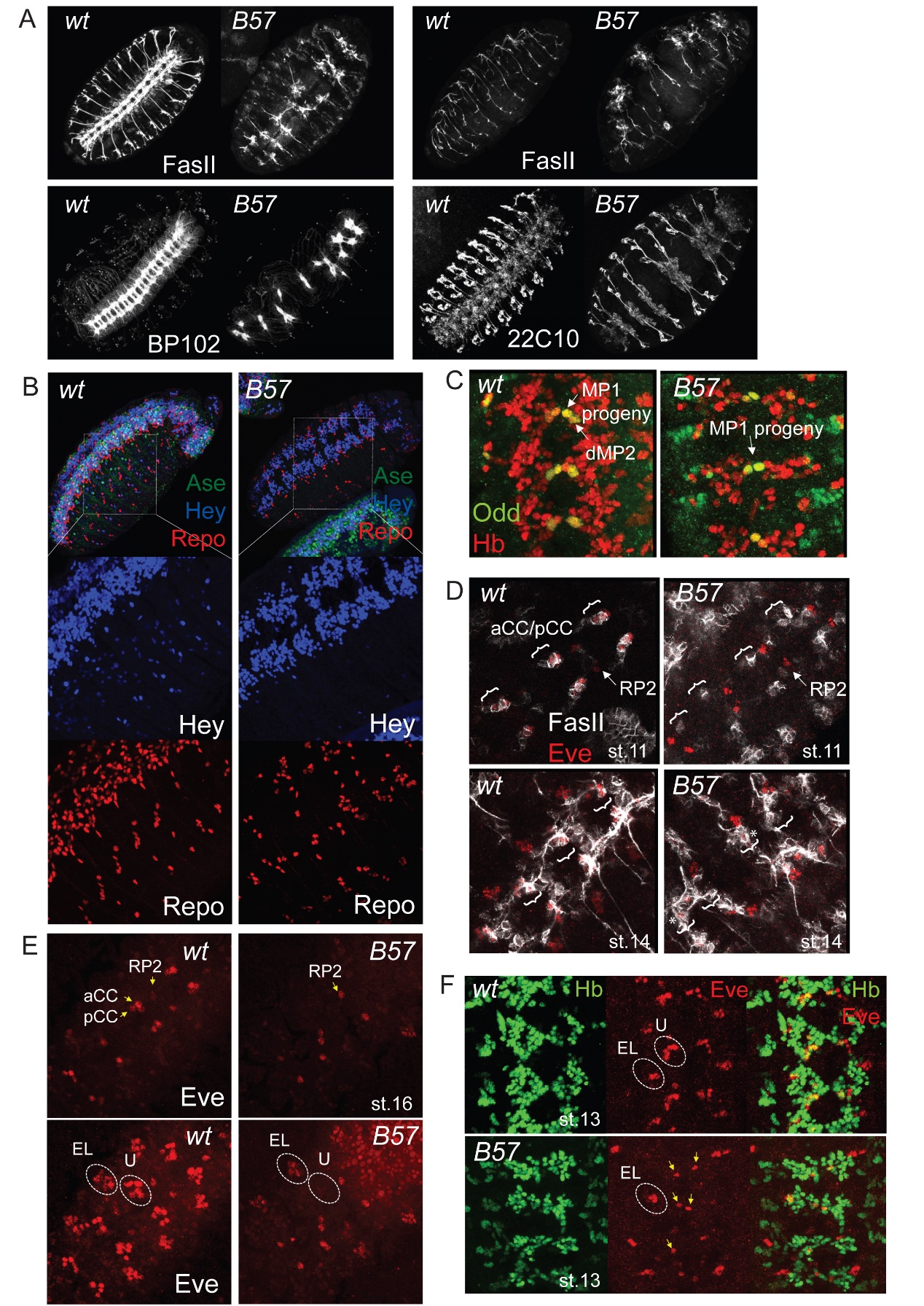


**Fig. S6. Depletion of the glia and neuronal populations in ASC mutants results in severe axonal defects.** A) Stage 16 Df(1)scB57 embryos, stained with FasII, BP102 and Futsch/22C10 display extensive neuronal hypoplasia. Note the complete lack of the longitudinal VNC tracts (FasII and BP102 ventral views, left panels), the defects in intersegmental and the segmental nerve development and pathfinding (FasII sagittal, top right panels) and the tightly apposed bilateral neuronal populations in the VNC neuromeres suggestive of incomplete midline function and production of repulsion cues (22C10 ventral view, bottom right panels). B) Repo (glia) and Hey (early neuron subset) staining reveal a severe loss in differentiated cell populations. C) The vMP2/dMP2 progeny of MP2 can not be identified with Odd/Hb in stage 13 embryos. dMP2 is normally Hb/Odd-positive, whereas vMP2 is Hb-positive, but has turned off Odd. In mutants only the MP1 progeny can be seen. D) The progeny of NB1-2, the aCC/pCC neurons are misspecified in B57 embryos. aCC/pCC are normally Eve/Fas2-positive, whereas RP2 is Eve-positive/ Fas2-negative at st.11. Bottom panels are from st.14 embryos, when more wt neurons have turned on Fas2. Note that the wt has started forming longitudinal tracts, whereas the mutant has not. E) At st.16 the Eve positive EL and U neurons are diminished in Dfsc(1)B57 embryos. The top and bottom panels are images from dorsal (top) and ventral (bottom) levels of the nerve cord of the same embryo F) St.13 embryos contain a reduced number of Eve-positive/ Hb-negative EL neurons and a single Hb/Eve-positive cell towards the midline, most probably the RP2 neuron.

**
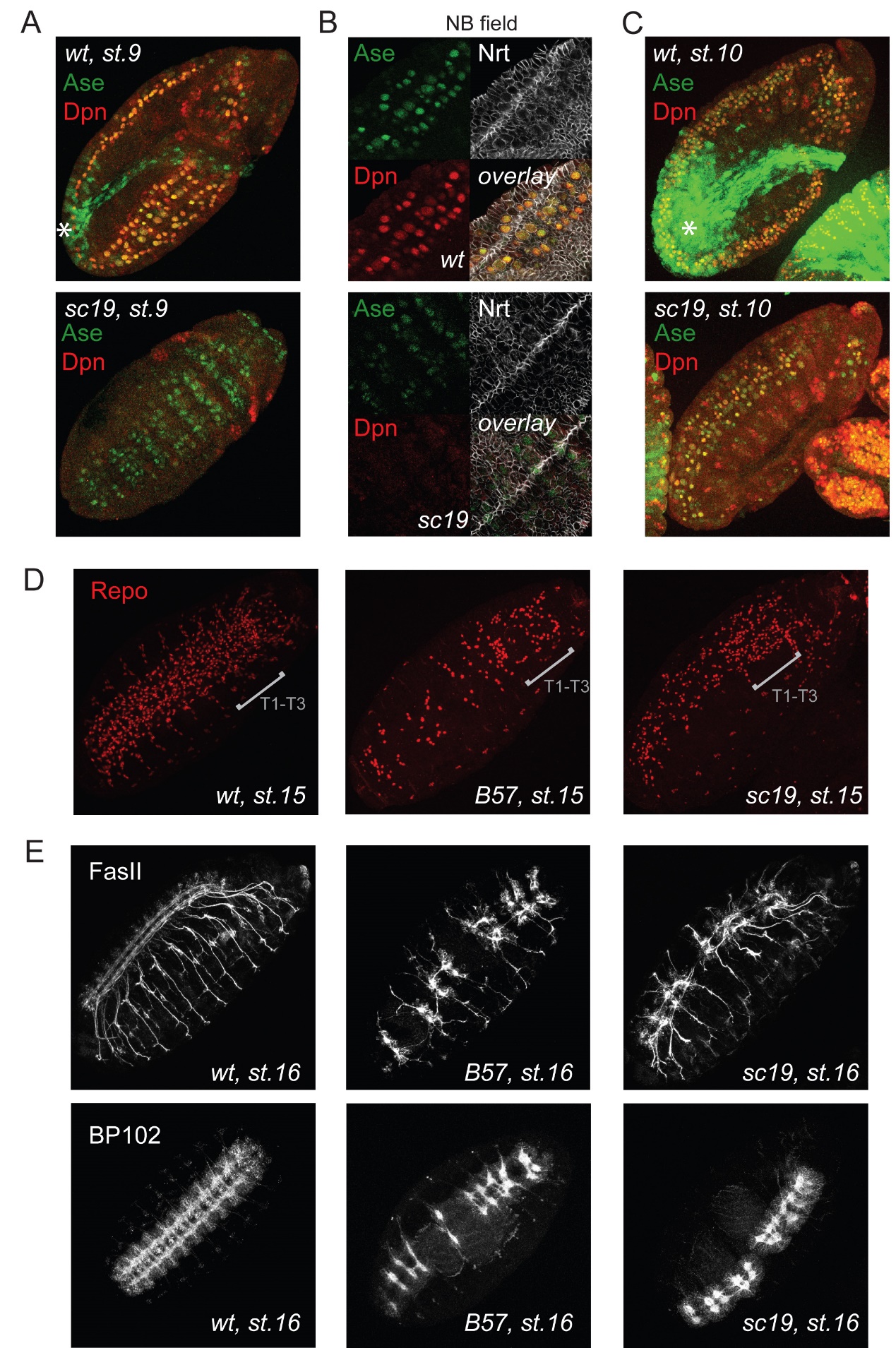
**

**Fig. S7. Asense provides partial neuroblast functionality and CNS development in the absence of ac, sc and l(1)sc.** A) Stage 9 wt and Df(1)sc19 embryos. Wt neuroblasts express Ase and Dpn robustly while mutant have weak transient expression of Ase at the NE layer. B) Single sections at the neuroblast level show that in Df(1)sc19 embryos not all neuroblasts (large round cells) express Ase and none express Dpn. Nrt marks cell outlines. C) During early stage 10 both wt and Df(1)sc19 embryos express both Dpn and Ase in neuroblasts. Note that the wt embryo also shows KrGal4>GFP expression (used to distinguish wt from mutant embryos) in the green channel, noted with a *. C) Df(1)sc19 embryos have more glia than Df(1)scB57 E) Axonal hypoplasia is less severe in Df(1)sc19 compared to Df(1)scB57. Overlay is the composite of three channels.

**
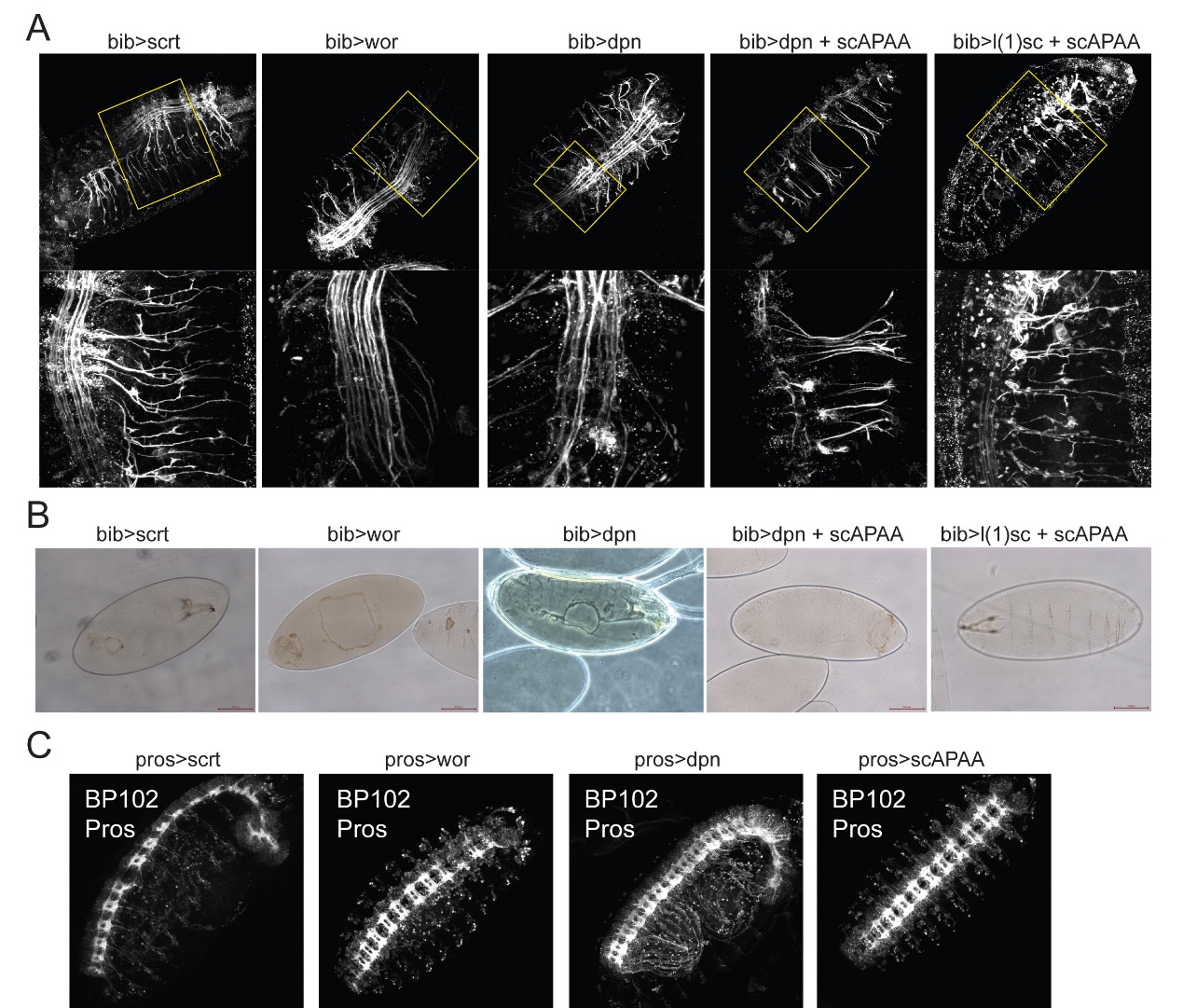
**

**Fig. S8. Neuroectodermal induction of proneural targets enhances neurogenesis at the expense of epidermal fate.** A) Neuroectodermal overexpression using bibGal4 of UAS-scrt, UAS-wor, UAS-dpn, UAS-dpn + UAS-scAPAA and UAS-l(1)sc + UAS-scAPAA result in highly hyperplastic late CNS phenotypes. A) Cuticle preparation from bibGal4 UAS-scrt, UAS-wor, UAS-dpn and UAS-dpn + UAS-scAPAA show ventral/ cephalic holes indicative of defects in the epidermis. C) Induction of UAS targets in the neuroblasts using pros-Gal4 does not affect late CNS development.
