# Supplementary material for: ASC proneural factors are necessary for chromatin remodeling during neuroectodermal to neuroblast fate transition to ensure the timely initiation of the neural stem cell program
Source: BMC Biol. 2022 May 13;20:107. doi: 10.1186/s12915-022-01300-8 (PMC9102361; doi:10.1186/s12915-022-01300-8)
Supplement: Supplementary file 3 — Additional file 3: Supplemental Methods. Fly strains, primers, antibodies, ChIP-seq protocol for low Drosophila embryo number, Ion Torrent Sequencing and Illumina sequencing. [file 12915_2022_1300_MOESM3_ESM.docx]

**Additional file 3: Supplemental Methods**

**Fly strains**

|  | FULL NAME | ORIGIN |
| --- | --- | --- |
| bib-GAL4 | P{GMR60F01-GAL4}attP2 | BDSC 39253 |
| pros-GAL4 | third chromosome homozygous viable P[w^+^] insertion | C. Gonzalez lab (IRB, Barcelona) |
| elav-Gal4 | P{GawB}elavC155 | BDSC 458 |
| lacZ reporters transgene recipient strain | y w nos-int ; attP40[y+] / (CyO) | BDSC 79604 |
| UAS-CD8-GFP | P{UAS-mCD8::GFP.L}LL4 | BDSC 5136 |
| UAS-scAPAA | UAS-myc-scAPAA | Delidakis lab; ([1](#_ENREF_1)) |
| UAS-NΔecd | P{UAS-Dl::N.ΔECN} | Giniger lab (NIH, Bethesda); ([2](#_ENREF_2)) |
| UAS-l(1)sc | UAS-myc-l(1)sc | this study |
| UAS-dpn | UAS-HA-dpn (lab id: U-8.1.1, on II) | Delidakis lab; ([3](#_ENREF_3)) |
| UAS-wor |  | FlyOrf F000155 |
| UAS-scrt |  | FlyOrf F001921 |
| UAS-Oli |  | FlyOrf F000021 |
| UAS-tap |  | FlyOrf F000103 |
| UAS-ase | UAS-ase (II) | Modolell lab (CBMSO, Madrid) |
| Df(1)sc-B57 | Df(1)sc-B57 | derived from BDSC 36560 |
| Df(1)sc^19^ | Df(1)sc^19^ | derived from BDSC 835 |
| FM7,KrG4>GFP | FM7c, P{GAL4-Kr.C}DC1, P{UAS-GFP.S65T}DC5, sn+ | BDSC 5193 |
| UAS-FUCCI | w[1118]; Kr[If-1]/CyO, P{ry[+t7.2]=en1}wg[en11]; P{w[+mC]=UAS-GFP.E2f1.1-230}26 P{w[+mC]=UAS-mRFP1.NLS.CycB.1-266}17/TM6B, Tb[1] | BDSC 55122 |
| esgGFP | PBac{fTRG10170.sfGFP-FT}VK00033 | VDRC 318400 |
| scrtGFP | PBac{fTRG00220.sfGFP-TVPTBF}VK00033 | VDRC 318073 |
| snaGFP | PBac{sna-GFP.24.8} | Stathopoulos lab (Caltech); ([4](#_ENREF_4)) |
| KrGFP | PBac{Kr-GFP.FPTB}VK00033 | BDSC 56152 |
| nvyGFP | PBac{fTRG01374.sfGFP-TVPTBF}VK00033 | VDRC 318347 |
| nerfin1GFP | P{nerfin-1-GFP.FPTB}attP40 | BDSC 67385 |
| tapGFP | P{tap-GFP.FPTB}attP40 | BDSC 68188 |
| OliGFP | PBac{Oli-GFP.FPTB}VK00033 | BDSC 68192 |
| MirrGFP | P{mirr-GFP.FPTB}attP40 | BDSC 68183 |
| OddGFP | PBac{odd-GFP.FPTB}VK00033 | BDSC 65290 |
| KrGFP | PBac{Kr-GFP.FPTB}VK00033 | BDSC 56152 |
| Df(1)260-1 | Df(1)260-1, y/FM4, rebalanced over FM7, Kr>GFP | BDSC 722 |

**Primers**

UAS-l(1)sc cloning: F: AAAACTCTCGCAAGGATTACC and R:CACTCACTAAGGCAACAATTTAGG

Enhancer cloning:

insc-KV1 (449 bp), F: TTATCAACATGGTGCGCTCT, R: AAGTTTCGGGTAATCTTATCAC

phyl-KV4 (534 bp), F: AATGAGCATCCAGCCAGAGG, R: TGGAGCACACCTTTTCAGC

nvy-KV14 (518 bp), F: CTGCTCTGAAGACCGAAGG, R: GATTGAACTGCACAACTGAGG

nvy-KV15 (454 bp), F: CGTCTCTGAGAGGCAATAGG, R: GGAAGCGAAAAGGAGATGG

brat-KV8 (588 bp), F: TGCAACACTTTCAGAATTTCC, R: GTTCATTAGCCGACCAAACG

scrt-KV19 (318 bp), F: ACGAGTTCTTGTCCCAGTCC, R: TGATAATGATGATTTGCTGATGC

scrt-KV10 (483 bp), F: TGGTACTCTCGTGCTGTTTCCACT, R: CTTGGCCGAATCTAAAATGC

tap-KV21 (492 bp), F: AGTAGCCAAAGCAAACAACC, R: CACCTTTTTACCCTTTTCTCG

dpn-KV23 (518 bp), F: ACCTAATCTCCCCGCAACC, R: GAACGTTTTAAGCTGATATCTATTCC

wor-KV29 (593 bp), F: CTCCGAATAGCACAGAAAGC, R: AAAGGATCTTGGCGTCTACC

**Antibodies**

Antibodies: Ase (rabbit, Jan lab, UCSF; guinea pig Knoblich lab, IMBA Vienna), Dpn (guinea-pig, Delidakis lab; Magadi et al 2020), Wor (rat, Abcam, ab196362), Hb (rabbit, Jaeckle lab; MPI, Goettingen), ph3 (rabbit, Abcam, ab5176), Pros (DSHB, MR1A), Nrt (DSHB, BP106), Elav (rat, DSHB, 7E8A10), BP102 (DSHB), FasII (DSHB), 22C10 (DSHB, Futsch**)**, Eve (DSHB 3C10), En (DSHB ,4D9), β-galactosidase (rabbit Cappel Labs) Mira (guinea-pig, Matzuzaki lab, RIKEN CDB, Kobe), GFP (rabbit, Minotech, 701-1), Repo (DSHB, 8D12), Hey (guinea-pig, Monastirioti lab IMBB, Heraklion; ([5](#_ENREF_5)). Mouse, rabbit, guinea pig or rat secondary antibodies conjugated to Alexa 488, 555, 568, 633 or 647 (Molecular Probes/ Thermo Fisher) or to FiTC, Cy3 or Cy5 (Jackson ImmunoResearch). H3K27Ac (Abcam, ab4729). Myc-epitope (rabbit, SantaCruz, sc-789, A-14). All DSHB antibories are mouse monoclonal antibodies (unless otherwise stated) and were obtained from the Developmental Studies Hybridoma Bank, created by the NICHD of the NIH and maintained at The University of Iowa, Department of Biology, Iowa City, IA 52242. anti-rabbit Grh was a kind contribution from Sarah Bray.

**ChIP-seq protocol for low Drosophila embryo number for Ion Torrent Sequencing**

-First day of ChIP

ChIP-seq experiments were performed with the protocol from ([6](#_ENREF_6)) adjusted to Drosophila embryos. Briefly, antibodies were bound on beads 80 ul paramagnetic Protein G beads with 8 μgr antibody sc-789 c-Myc (A-14). For the H3K27Ac ChIP-seq we used 60 μl of Protein G beads to pre-bind 6 μgr of Histone H3 (acetyl K27) from Abcam (ab4729) overnight at 4^o^C.

-Second day of ChIP

Appropriate volumes of 1 X PBS/0.1 Triton-X, 1 X PBS, LB1, LB2 and LB3 buffers were chilled on ice. The clean 7 ml Wheaton glass homogenizer was placed in ice as well. All centrifugation steps were performed on a pre-chilled to 4^o^C benchtop centrifuge. Embryo collections tubes were removed from -80^o^C and left to thaw on ice with mild finger tapping for embryos to become loose. For each ChIP-seq experiment, 10 embryo collections from the above-mentioned fly cages were pooled to a 7 ml Wheaton glass homogenizer using PBS/T for transferring with glass Pasteur pipettes. Embryos were allowed to sink and subjected to a second 2 ml cold PBS rinse. We added 1 ml PBS and gently dounced embryos with the tight plunger for approximately 10 strokes until embryo dissociation, without visible whole embryo clumps. Homogenized tissue was poured in 1.5 ml collection eppendorfs and spun at 7.000 rpm for 7 minutes. PBS was removed and the tube bottoms gently tapped to detached cell pellets. Cells were resuspended in 1.5 ml LB1 by mild pipetting and transferred to a cold room to rotate gently for 10 minutes on a wheel. Spun at 8.000 rpm for 5 minutes. Discarded LB1, flicked nuclear pellet to loosen and added 1.5 ml LB2 for a second mild wash to remove cytoplasmic remnants. Pipetted a couple of times up and down gently. Left rotating in cold room for 5 min. Spun at 8000 rpm for 5 min. Discarded LB2, and the nuclear pellet was resuspended in 600 ul LB3. Pipetted again gently for a couple of times and split in two 1.5 ml ependorfs. Sonicated nuclei for 8-10 cycles, 30’’ on 30’’ off, in a Bioruptor®Plus (Diagenode). Added 30 μl of 10% Triton-X to each 300 μl sonicated chromatin, mixed by pipetting and spun at max 13-14k rpm for 10 minutes. Transferred the two 300 μl of chromatin in one fresh tube without taking the pellets and left on ice. Kept 20 μl of the chromatin as input and froze in -20^o^C. Washed the antibody-bound beads twice with PBS and finally resuspended them in 300 μl of LB3. Transferred the 300 μl bead suspension to the 600 μl chromatin, mixed gently to wash out beads from tip and transferred tubes to the cold room to rotate overnight.

-Third and fourth days of ChIP

Followed the ([6](#_ENREF_6)) protocol and at the end resuspended ChIP DNA in 25 μl DNAse free water. Kept ChIP DNA in -20^o^C until library preparation. In case of qPCR verification prior to library preparation diluted 2 μl of ChIP DNA in 40 μl final volume and used 4 μl for each qPCR reaction using SYBR green at 12 μl final reaction. The qPCR step was used to evaluate enrichment efficiency.

Libraries were prepared according to manufacturer’s protocol using the Ion Plus Library Kit (# 4471252) and sequenced on the Ion Torrent Proton platform using Ion 540 ChIP kits.

**Illumina sequencing.** The H3K27Ac ChIP-seq biological replicate 2 was performed on Illumina Nextseq500 on 1x75 single flowcell. Libraries were made using the NEB next Ultra II DNA kit for Illumina. ChIP material was performed as described above with the only exception that sonication was performed with 2 sequential rounds of 5 cycles each (30’’on High/30’’off) with intermediate brief spin of the samples and addition of ice in the Bioruptor’s sonication water bath.

**References**

1. Kiparaki M, Zarifi I, Delidakis C. bHLH proteins involved in Drosophila neurogenesis are mutually regulated at the level of stability. Nucleic acids research. 2015;43(5):2543-59.

2. Fuerstenberg S, Giniger E. Multiple roles for notch in Drosophila myogenesis. Developmental biology. 1998;201(1):66-77.

3. Magadi SS, Voutyraki C, Anagnostopoulos G, Zacharioudaki E, Poutakidou IK, Efraimoglou C, et al. Dissecting Hes-centred transcriptional networks in neural stem cell maintenance and tumorigenesis in Drosophila. Development. 2020;147(22).

4. Dunipace L, Ozdemir A, Stathopoulos A. Complex interactions between cis-regulatory modules in native conformation are critical for Drosophila snail expression. Development. 2011;138(18):4075-84.

5. Monastirioti M, Giagtzoglou N, Koumbanakis KA, Zacharioudaki E, Deligiannaki M, Wech I, et al. Drosophila Hey is a target of Notch in asymmetric divisions during embryonic and larval neurogenesis. Development. 2010;137(2):191-201.

6. Schmidt D, Wilson MD, Spyrou C, Brown GD, Hadfield J, Odom DT. ChIP-seq: using high-throughput sequencing to discover protein-DNA interactions. Methods. 2009;48(3):240-8.
